# Human ALS/FTD brain organoid slice cultures display distinct early astrocyte and targetable neuronal pathology

**DOI:** 10.1038/s41593-021-00923-4

**Published:** 2021-10-21

**Authors:** Kornélia Szebényi, Léa M. D. Wenger, Yu Sun, Alexander W. E. Dunn, Colleen A. Limegrover, George M. Gibbons, Elena Conci, Ole Paulsen, Susanna B. Mierau, Gabriel Balmus, András Lakatos

**Affiliations:** 1 John van Geest Centre for Brain Repair, Department of Clinical Neurosciences, University of Cambridge, Cambridge Biomedical Campus, Cambridge, UK; 2 UK Dementia Research Institute, Cambridge Biomedical Campus, Cambridge, UK; 3 Department of Clinical Neurosciences, University of Cambridge, Cambridge Biomedical Campus, Cambridge, UK; 4 Department of Physiology, Development and Neuroscience, University of Cambridge, Cambridge, UK; 5 Wellcome Trust–MRC Cambridge Stem Cell Institute, Cambridge Biomedical Campus, Cambridge, UK

**Keywords:** Amyotrophic lateral sclerosis, Neurological models, Induced pluripotent stem cells

## Abstract

Amyotrophic lateral sclerosis overlapping with frontotemporal dementia (ALS/FTD) is a fatal and currently untreatable disease characterized by rapid cognitive decline and paralysis. Elucidating initial cellular pathologies is central to therapeutic target development, but obtaining samples from presymptomatic patients is not feasible. Here, we report the development of a cerebral organoid slice model derived from human induced pluripotent stem cells (iPSCs) that recapitulates mature cortical architecture and displays early molecular pathology of *C9ORF72* ALS/FTD. Using a combination of single-cell RNA sequencing and biological assays, we reveal distinct transcriptional, proteostasis and DNA repair disturbances in astroglia and neurons. We show that astroglia display increased levels of the autophagy signaling protein P62 and that deep layer neurons accumulate dipeptide repeat protein poly(GA), DNA damage and undergo nuclear pyknosis that could be pharmacologically rescued by GSK2606414. Thus, patient-specific iPSC-derived cortical organoid slice cultures are a reproducible translational platform to investigate preclinical ALS/FTD mechanisms as well as novel therapeutic approaches.

## Abstract (web summary)

By developing a long-term ALS/FTD patient-specific iPSC-derived organoid model that recapitulates mature cortical cell types, the authors pinpoint early selective molecular pathologies at single-cell resolution and a druggable neuronal vulnerability.

## Main

Cerebral organoids represent a promising tool for understanding human brain physiology and disease processes^1^. Their resemblance to the human cerebral cortex—in terms of their three-dimensional (3D) architecture, cell-type diversity and cell–cell interactions^2,3^—provides major advantages over other stem-cell-derived culture and mouse models. Organoids provide a suitable biological platform for assessing dynamic sequences of human-specific cellular events, especially those relevant to neurodegenerative disease research, in which many cell types are now implicated in pathogenesis. However, the timing and cell-specific characteristics of molecular disturbances remain unclear^4^. Such investigations could help treatment strategies aiming to prevent pathological triggers. Since the experimental use of samples from presymptomatic human patients is not feasible, cerebral organoid technologies raise the possibility of capturing the initiating cell-type-specific pathologies of neurodegenerative diseases.

Despite recent technical improvements in organoid culture methods^5–7^, there are a number of challenges remaining for neurodegenerative disease modeling. Brain organoids have been grown from cells derived from patients with Parkinson’s disease and from patients with Alzheimer’s disease for 30 or 84 days in vitro (DIV), respectively^8,9^. This has been a considerable step forward in disease model development; however, the relatively short longevity and variable cortical-cell-type composition^10,11^ may limit the fidelity for observing a broad spectrum of pathology. In addition, nutrient and oxygen supply^12^ is a particular problem in non-vascularized organoids, restricting growth and viability and therefore potentially the final steps of cell differentiation and maturation. Recent reports suggest that slicing brain organoids derived from embryonic stem cells and/or cultivating them on fenestrated membranes improve viability and potentially cell composition^5,6^. Similar advances are a prerequisite for patient-specific iPSC-derived organoids for precise pathomechanistic discoveries. This is particularly warranted for ALS/FTD, an untreatable neurodegenerative disease with rapid cognitive decline and paralysis.

Here, we report the development of a long-term human cortical organoid (CO) model that closely recapitulates early molecular pathology of ALS/FTD. COs were grown up to 240 DIV at the air–liquid interface (ALI-COs) from iPSCs derived from patients with ALS/FTD, harboring the *C9ORF72* hexanucleotide repeat expansion mutation (C9 ALI-COs). This mutation is useful for ALS/FTD modeling as it gives rise to a wide range of pathologies in both sporadic and inherited disease forms^13^. Using 587 ALI-CO slices cultured from human iPSC (hiPSC)-derived organoids, we show that ALI-COs develop consistent microarchitecture and mature cortical-circuit-forming disease-relevant phenotypes. Furthermore, we found that C9 ALI-COs, although lacking microglia and vasculature, exhibit astroglia- and neuron-specific disturbances. These include distinct transcriptional and proteostasis disturbances in astroglia and neurons, including early accumulation of the autophagy signaling protein P62 and the toxic dipeptide repeat protein (DPR) poly(GA), respectively. Deep layer neurons (DLNs) display DNA damage and cell death that we pharmacologically rescued by improving proteostasis. Our results demonstrate that hiPSC-derived ALI-COs provide a reproducible platform with the necessary longevity and maturity for investigating ALS/FTD, thereby revealing early and targetable cell vulnerabilities^4,14^ relevant to presymptomatic clinical stages.

## Results

### Diverse cell populations segregate in hiPSC-derived ALI-COs

**Fig. 1:**
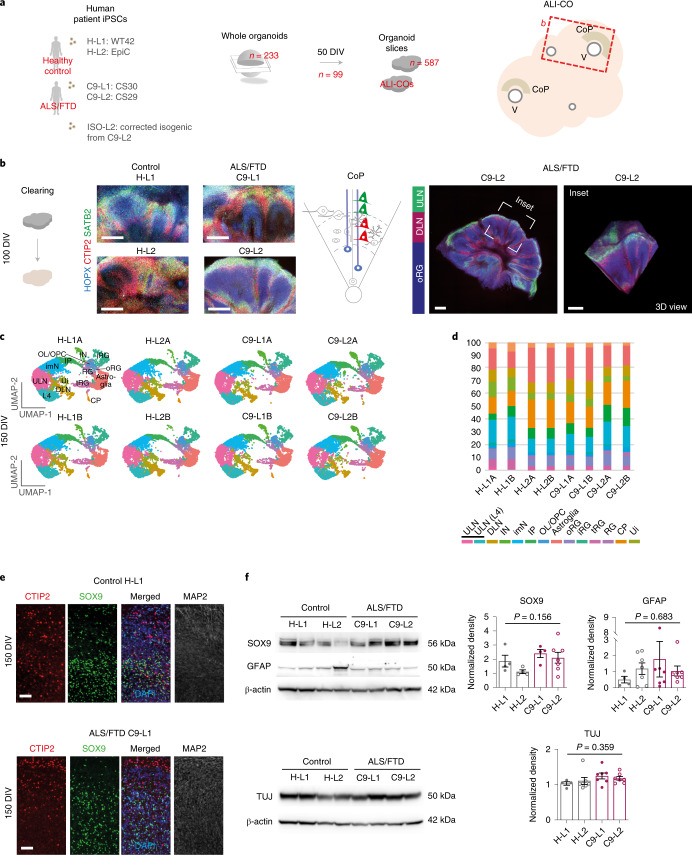
Cortical plate cytoarchitecture is recapitulated in C9 and control ALI-COs. **a**, Schematic of cell line sources and generation of ALI-COs displaying ventricle-like (V) and cortical plate (CoP) formation. **b**, Projected 8-plane *z*-stack (left), single 2D plane (right) and 3D (inset) views of confocal immunofluorescence images of cleared ALI-COs (right) at 100 DIV, which demonstrate layers (schematic) of HOPX^+^ oRG (cyan), CTIP2^+^ DLNs (red) and SATB2^+^ ULNs (green). **c**, Uniform manifold approximation and projection (UMAP) plots represent 14 color-coded clusters identified in the scRNA-seq dataset (*n* = 75,497 cells) for all control and C9 ALI-COs at 150 DIV using cell-marker genes^15^. ULN, upper layer cortical neuron; L4, layer 4 ULN; DLN, deep layer neuron; IN, interneurons; imN, immature neurons; IP, intermediate progenitors; OL/OPC, oligodendrocyte lineage/progenitor cells; RG, radial glia; oRG, outer RG; iRG, inner RG; tRG, truncated RG/stressed RG; CP, choroid plexus; Ui, unidentified. **d**, Bars represent color-coded cluster identity distributions expressed as cell proportions in each pooled ALI-CO pair sample. **e**, Representative images of immunostained ALI-COs at 150 DIV for 3 biological replicates per group displaying CTIP2^+^ DLN (red) and SOX9^+^ astroglia (green) with or without DAPI stain (blue) and MAP2^+^ (gray) neurons. **f**, Representative WB images (left) for SOX9, GFAP, TUJ and β-actin in samples of ALI-COs at 150 DIV and quantified (right) SOX9, GFAP and TUJ WB band density levels normalized to β-actin. Data indicate the mean ± s.e.m.; *n* = 4, 4, 4, 7 (SOX9) and 4, 7, 7, 7 (GFAP/TUJ) independent ALI-COs for H-L1, H-L2, C9-L1, C9-L2 organoid lines, respectively; one-way ANOVA with Tukey’s post hoc test (overall ANOVA *P* values are indicated in the graphs). Scale bars, 40 μm (**e**), 50 μm (**b**, left) and 500 μm (**b**, right). See Supplementary Table 3 for detailed statistics and source data for unprocessed WB images. Source data

**Extended Data Fig. 1:**
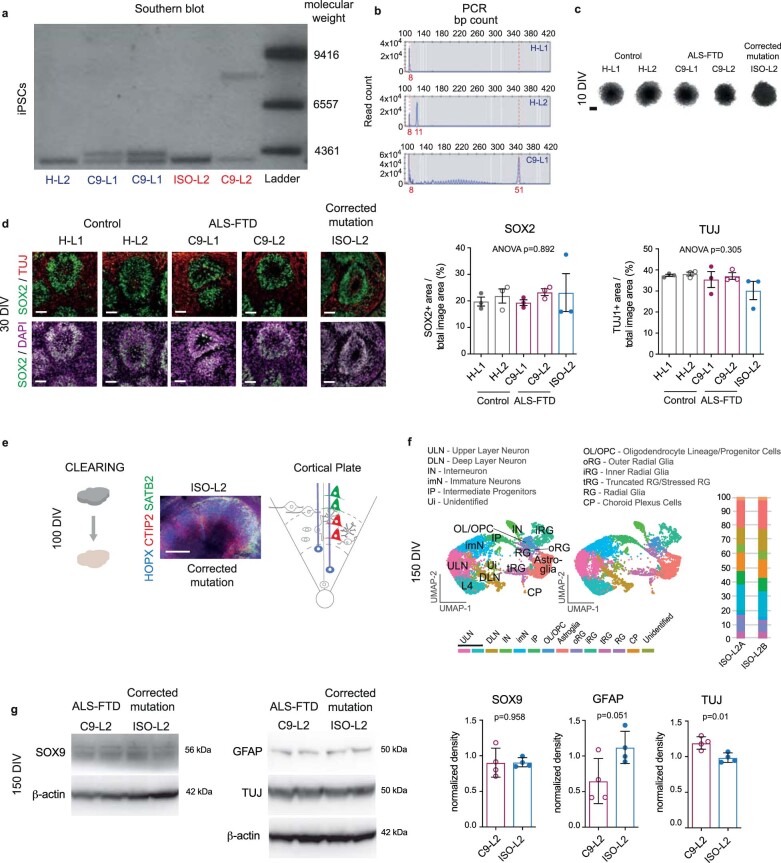
Human iPSC genotyping and consistent early organoid formation. **a**, Southern blot demonstrates C9ORF72 hexanucleotide repeats in C9-L2 iPSCs and its absence in the isogenic mutation-corrected control line, ISO-L2. Shorter repeat length is shown for C9-L1 iPSCs versus the C9-L2 lines.﻿ **b**, PCR-based fragment analysis of iPSC lines with shorter repeat length. **c**, Expanded neuroepithelia show similar bud-formation between COs at 10 DIV. **d**, Representative immunofluorescence images of COs demonstrating palisade-like organization of SOX2^+^ progenitor cells (green) surrounded by immature TUJ^+^ neurons (red) in COs at 30 DIV and quantification of SOX2 and TUJ immunoreactivity. Data expressed as mean ± s.e.m.; n = 3 independent COs per group; One-way ANOVA with Tukey’s post-hoc test (overall p-values are indicated in graphs). **e**, Projected 8-plane z-stack views of confocal immunofluorescence images of cleared ISO-L2 ALI-CO at 100 DIV, demonstrating layers (schematic) of HOPX^+^ outer radial glia (cyan), CTIP2^+^ DLNs (red) and SATB2^+^ upper layer neurons (green). **f**, UMAP plots represent 14 color-coded clusters identified in the scRNA-seq dataset (n = 75,497 cells from 4 ISO-L2 ALI-COs at 150 DIV), using cell-marker genes^15^. Bars represent color-coded cluster identity distributions expressed as cell proportions in each pooled ALI-CO-pair sample. ULN: upper layer cortical layer neurons; L4: layer 4 ULN; DLN: deep layer cortical layer neurons; IN: interneurons; imN: immature neurons; IP: intermediate progenitors; Astroglia: mature and developing astrocytes; OL/OPC: oligodendrocyte lineage/oligodendrocyte progenitor cells; oRG: outer RG; iRG: inner radial glia; tRG/stress: ‘truncated’ radial glia or stressed RG; RG: radial glia; CP: choroid plexus. **g**, Representative western blots for SOX9, GFAP, TUJ and β-actin in samples of ALI-COs at 150 DIV and quantification of SOX9, GFAP and TUJ band density levels normalized to β-actin. Data indicate mean ± s.e.m.; n = 4 independent C9-L2 and ISO-L2 ALI-COs; Two-tailed unpaired t-test. Scale bar=300 μm for c, 50 μm for d, 500 μm for e. See Supplementary Table 3 for detailed statistics and Source Data for unprocessed WB images. Source data

**Extended Data Fig. 2:**
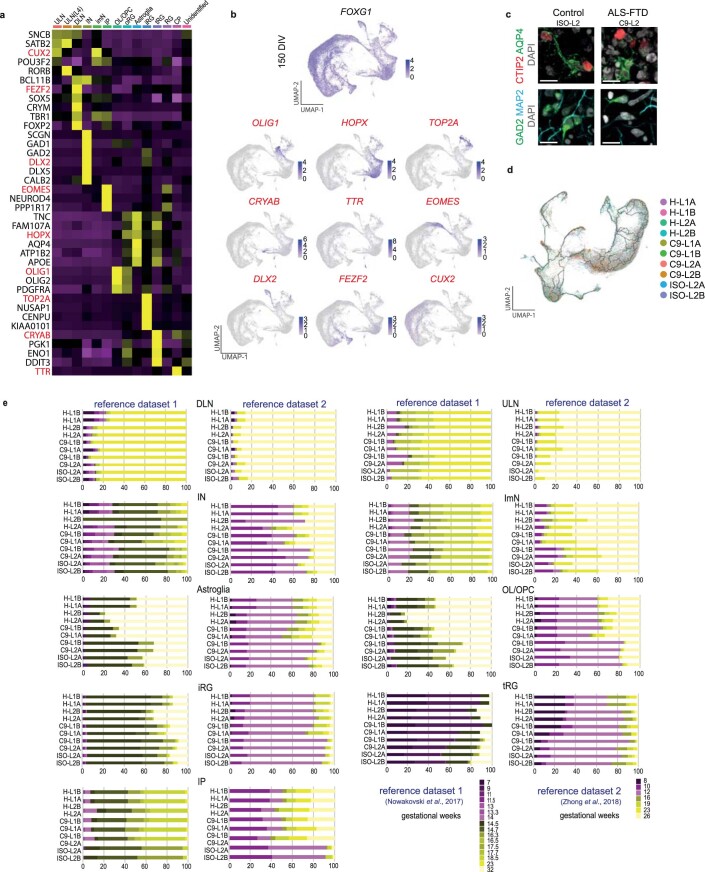
Cortical cell-subtype transcriptional maturity profiles are recapitulated in control and C9 ALI-COs at 150 DIV. **a**, Average gene expression of marker genes for various subpopulations identified in 14 unbiased clusters by scRNA-seq within the merged dataset of control and C9 ALI-COs (n = 75,497 cells). ULN: upper layer cortical neurons; ULN(L4): layer 4 neurons; DLN: deep layer cortical neurons; IN: interneurons; imN: immature neurons; IP: intermediate progenitors; Astroglia: mature and developing astrocytes; OL/OPC: oligodendrocyte lineage/oligodendrocyte progenitor cells; oRG: outer RG; iRG: inner radial glia; tRG/stress: ‘truncated’ radial glia or stressed RG; RG: radial glia; CP: choroid plexus. **b**, UMAP representations of scaled expression levels and distribution of key marker genes (in red) of major cell-types. **c**, Representative immunofluorescence images of CTIP2^+^ DLNs, GAD2^+^ INs and AQP4^+^ astroglia for 3 biological repeats. **d**, UMAP representation of inferred developmental trajectories in control and C9 ALI-COs, obtained by using Monocle3. **e**, Bar plots indicate proportion of cells mapping to transcriptional profiles of various fetal ages^16,17^ in scMAP^18^. Scale bar=10 μm for c.

First, we examined whether C9 ALI-COs develop similar cell-type diversity and cytoarchitecture to their healthy control patient-derived counterparts to assess their relevance to disease-affected phenotypes. The following ALI-COs were produced: fibroblast-derived (H-L1) and cord-blood-derived (H-L2) control hiPSC lines; two fibroblast-derived C9 ALS/FTD hiPSC lines with ~50 (C9-L1) or ~1,000 (C9-L2) hexanucleotide repeat expansions; and a mutation-corrected isogenic (ISO-L2) line (Fig. 1a, Supplementary Table 1 and Extended Data Fig. 1a,b). The iPSC-derived ALI-COs were generated by adapting protocols for CO generation via embryonic stem cells^5^. Gross morphogenesis at 10 DIV and progenitor germinal zones at 30 DIV were comparable in control and C9 COs (Extended Data Fig. 1c,d and Supplementary Fig. 1a). Slicing COs at 50 DIV (Fig. 1a) and culturing at the ALI, to increase nutrition and longevity, supported consistent organoid specification to forebrain identity as marked by widespread FOXG1 immunoreactivity (Supplementary Fig. 1b). By 75 DIV, a distinct neuronal population arose with strong CTIP2 immunoreactivity, which characterizes DLNs in the developing cortex (Supplementary Fig. 1b). From 100 DIV onwards, SATB2^+^ upper layer cortical neurons (ULNs) spatially segregated from DLNs and HOPX^+^ radial glia (RG) (Fig. 1b). Despite moderate heterogeneity in the 3D morphology between ALI-COs, the microarchitecture and cell-type composition of cortical layers were consistently similar in all control and C9 ALI-COs regardless of the source of the cell line (Fig. 1b, Extended Data Fig. 1e and Supplementary Videos 1–5). For a comprehensive cell-subtype analysis, we characterized the single-cell transcriptomic profile of C9 versus control ALI-COs from all lines at 150 DIV using single-cell RNA-sequencing (scRNA-seq). Clustering was performed on merged non-batch-corrected samples for accurate comparison (Fig. 1c). We identified 13 out of the 14 distinct clusters, which translated to major cell-type identities based on established marker genes and one cell-state identity^15^ (Fig. 1c,d and Extended Data Figs. 1f,g and 2a–c). These all possessed forebrain identity signatures, and cell-type proportions were conserved between control and C9 ALI-COs (Fig. 1d, Extended Data Fig. 2a,b and Supplementary Fig. 1b,c). Glia and neuron composition was comparable in all ALI-COs, with mild variation mainly between the control lines (Fig. 1d and Extended Data Fig. 1f). This observation was reinforced by immunohistochemistry and western blots of whole organoid lysates, which showed similar protein levels of cell markers (that is, SOX9/GFAP and TUJ) for astroglia and neurons (Fig. 1e,f, Extended Data Fig. 1g and Supplementary Fig. 1c). In summary, control and C9 ALI-COs showed similar cortical morphogenesis and representation of major glial and neuronal cell types at 150 DIV.

### C9 ALI-COs recapitulate mature cortical cell subtypes

**Fig. 2:**
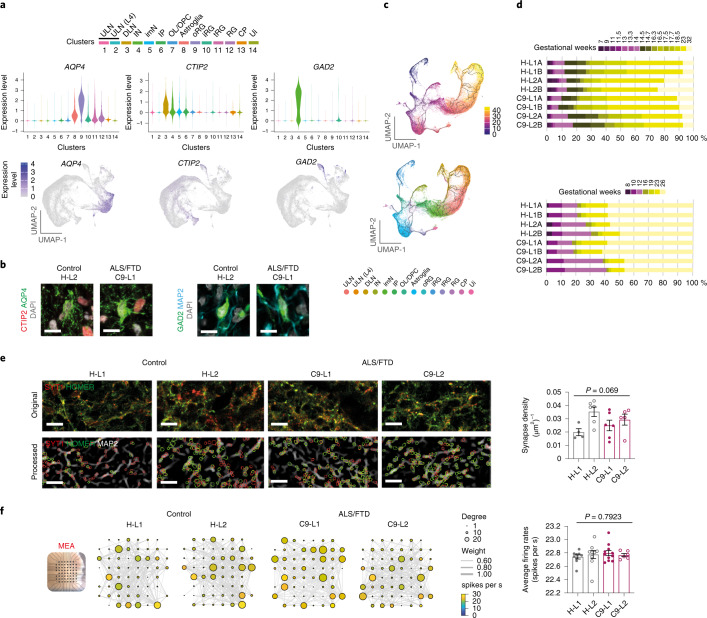
Cortical-cell-type diversity and maturity are recapitulated in C9 and control ALI-COs. **a**, Violin plots (upper) and UMAP plots (lower) illustrating *AQP4* (astrocyte), *CTIP2* (DLN) and *GAD2* (IN) marker gene expression identified by scRNA-seq in 20 ALI-COs at 150 DIV. Cluster numbers (*x* axis) correspond to color-coded cell-type identities as indicated above the plots. **b**, Representative images demonstrating the immunoreactivity of cell-type marker proteins in ALI-COs (three biological repeats per group). **c**, Pseudotime analysis of scRNA-seq data with branches representing the color-coded trajectory of cell-subtype specification (upper) or cluster identities (lower). *n* = 75,497 cells for **a** and **c**. **d**, Projected transcriptomic data of four control and four C9 ALI-CO slice-pairs onto two fetal brain reference datasets^16,17^ using scmap^18^. Bars indicate the proportion of cells assigned to different fetal ages and expressed as human gestational weeks. **e**, Representative original confocal microscopy images (upper) illustrate pre- and post-synaptic proteins, synaptotagmin-1 (SYT1; red) and HOMER1 (HOMER; green) and their colocalization over MAP2^+^ dendrites (gray) in deconvoluted digital images (lower) in ALI-COs at 150 DIV and quantification (right). Data represent the mean ± s.e.m. synapse densities; *n* = 4, 6, 6, 5 independent ALI-CO slices for H-L1, H-L2, C9-L1, C9-L2 organoid lines, respectively; one-way ANOVA with Tukey’s post hoc test (overall ANOVA *P* value is indicated in the graph). **f**, Neuronal network analysis using MEAs (left) reveals rich spontaneous network activity in all ALI-COs. Highly connected nodes (circles) represent individual electrodes for the degree of connectivity (node size), the spike rates (node color) and the connection strength (line thickness). The graph (right) shows the firing rates (spike number per second) across all electrodes for each recording. Data are expressed as individual recordings and the mean ± s.e.m.; *n* = 9, 9, 11, 6 ALI-CO slices for H-L1, H-L2, C9-L1, C9-L2 organoid lines, respectively; one-way ANOVA with Tukey’s post hoc test (overall ANOVA *P* value is indicated in the graph). Scale bar, 10 μm (**b**,**e**). See Supplementary Table 3 for detailed statistics.

Next, we examined whether the transcriptomic signatures revealed cell subtypes, including layer-specific cell populations involved in cortical circuits. We identified mature neuronal subpopulations including interneuron (IN; *GAD2* and *DLX2*), DLN (*CTIP2* and *FEZF2*) and ULN (*SATB2*, and *CUX2*) excitatory neurons. We also identified astrocytes or astroglia (*AQP4*), choroid plexus (CP) cells (*TTR*) and immature progenitor cells, including inner radial glia (iRG; *TOP2A*), outer radial glia (oRG; *HOPX*), oligodendrocyte lineage or progenitor cells (OL/OPCs; *OLIG1* and *PDGFRA*) and intermediate progenitors (IPs: *EOMES*) (Fig. 2a,b and Extended Data Figs. 1g and 2a–c). Notably, a *CRYAB*-expressing RG population shared a transcriptomic profile with previously described “truncated radial glia”^16^; however, its identity has equally reflected cells with increased expression of genes involved in glycolysis (Extended Data Fig. 2a,b). The cell-subtype-specific transcriptomic signatures indicated broad similarities in cell composition between control and C9 ALI-COs that are consistent with the immunohistochemical findings.

We next compared the single-cell transcriptomic maturity profiles of control and C9 ALI-COs with human brain tissue datasets. First, we carried out a pseudotime trajectory analysis using the merged control and C9 ALI-CO scRNA-seq dataset to predict temporal sequences of cell specification. Both neuronal and glial trajectories transitioned through RG and immature cell types toward mature DLN, ULNs, INs and astrocytes, as visualized by branches in the graphs (Fig. 2c). No differences were found between independent control and C9 ALI-COs in the reconstructed path recapitulating the milestones of human brain development (Fig. 2c and Extended Data Fig. 2d). To confirm this finding, we projected transcriptomic data obtained from the organoid cell populations onto age-specific reference datasets derived from 56- to 182-day-old fetal brains^16,17^ using scmap^18^. The maturity profiles of most cells in all ALI-COs projected similarly onto 161- to 182-day-old fetal brains, corresponding to 23–26 gestational weeks (Fig. 2d and Extended Data Fig. 2e). Taken together, our scRNA-seq-based computational analysis and immunohistochemical data support the recapitulation of cytoarchitecture- and age-matched forebrain development for control and C9 ALI-COs. While cortical cell-type diversity was consistent throughout, the ALI-COs lacked cells of mesodermic origin, such as microglia or blood vessels. This provides a unique opportunity to examine whether astroglial and neuronal subtypes are sufficient to recapitulate ALS/FTD-related molecular pathological hallmarks in C9 ALI-COs.

### Neuronal networks are functionally active in C9 ALI-COs

We addressed whether cortical cell diversity and maturity in control and C9 ALI-COs are also reflected in their ability to form functional networks. We identified synapses by applying a CellProfiler algorithm-based proximity analysis of co-immunolabeled pre- and post-synaptic proteins, synaptotagmin-1 (SYT1) and HOMER1 over MAP2^+^ dendritic areas in ALI-CO sections (Fig. 2e and Supplementary Fig. 2a). Synapse densities were similar between all C9 and control ALI-COs (Fig. 2e). To measure neuronal activity and functional connectivity in the ALI-COs, multielectrode array (MEA) recordings were performed using 3D MEA chips (Fig. 2f). At 150–193 DIV, ALI-COs derived from the C9 iPSC lines (C9-L1 and C9-L2) showed similar levels of spontaneous activity to those from control (H-L1 and H-L2) lines (Fig. 2f and Supplementary Fig. 2b–d). Functional connectivity was inferred from correlated spontaneous activity using the spike-timing tiling coefficient. Our graph-theory-based network analysis demonstrated a comparable connectivity pattern between control and C9 ALI-COs (Fig. 2f and Supplementary Fig. 2e–j). These findings show that control and C9 ALI-COs similarly recapitulate many structural and functional aspects of brain development. This enabled us to address early molecular pathology in somatic cells in C9 ALI-COs, which may already be present in embryonic or perinatal stages^19^.

### scRNA-seq reveals perturbed cell homeostasis in C9 ALI-COs

**Fig. 3:**
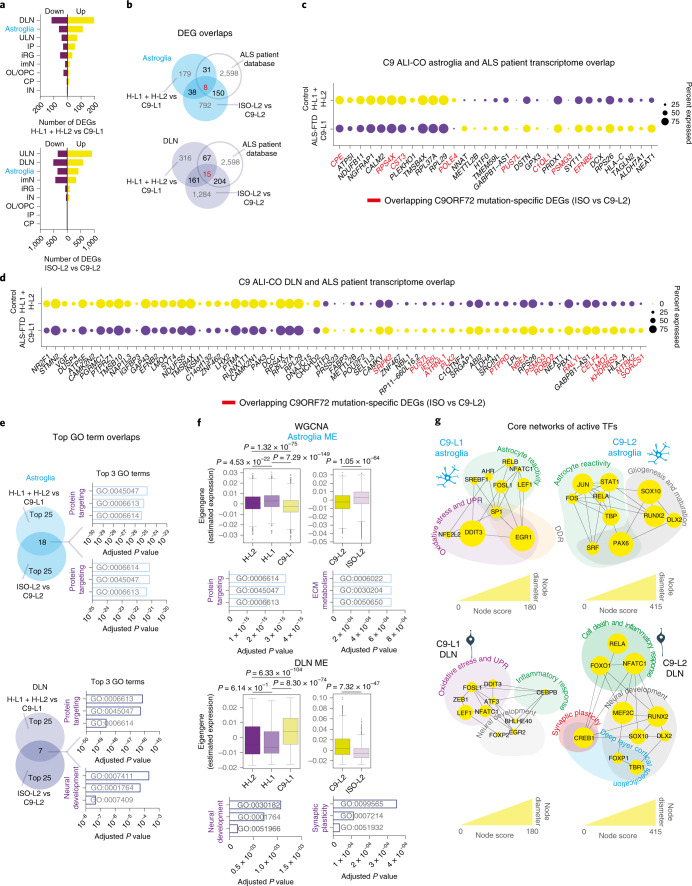
C9 ALI-COs at 150 DIV show similarities to ALS-related transcriptional profile, cell stress pathways and adaptive changes. **a**, Number of DEGs per cell type in C9-L1 versus H-L1/H-L2 control ALI-COs (upper) and C9-L2 versus mutation-corrected isogenic ISO-L2 control (lower) ALI-COs (four slices per organoid line). **b**, Venn diagrams display the number of overlapping genes between C9-L1 and C9-L2 ALI-CO DEGs and ALS-related transcriptomic changes. **c**,**d**, Dot plots represent the overlap in transcriptomic changes between astroglia (**c**) or DLNs (**d**) in control or C9 ALI-COs and samples from patients with ALS^20–25^. The dot size indicates the percent of cells expressing overlapping upregulated (yellow) and downregulated (purple) genes. Gene names in red indicate overlapping genes between C9-L1 ALI-CO and C9-L2 ALI-CO (mutation-specific) DEGs and ALS patient sample-related transcriptomic changes. **e**, Venn diagrams represent top astroglia- (upper) and DLN-related (lower) GO terms that overlap between C9-L1 ALI-CO and C9-L2 ALI-CO (mutation-specific) DEGs. **f**, Boxes, lines, whiskers display the quartile, median and minimum–maximum distribution (without the outliers) representing top differentially expressed module eigengenes (WGCNA) for astroglia and DLNs in two H-L2, 2 H-L1, 2 C9-L1, 2 C9-L2, 2 ISO-L2 independent ALI-CO slice-pairs (4 slices per line), respectively. Two-sided Mann–Whitney–Wilcoxon test with Bonferroni correction. Bar graphs show the corresponding top significantly enriched GO terms. **g**, Core interaction networks (STRING) of TFs with predicted activity (SCENIC) defined by the top 10 betweenness centrality score (Cytohubba in Cytoscape) for interacting TFs (nodes) and grouped into functional domains based on gene/protein function (https://www.uniprot.org). Node size illustrates centrality score. See Supplementary Table 3 for detailed statistics.

**Extended Data Fig. 3:**
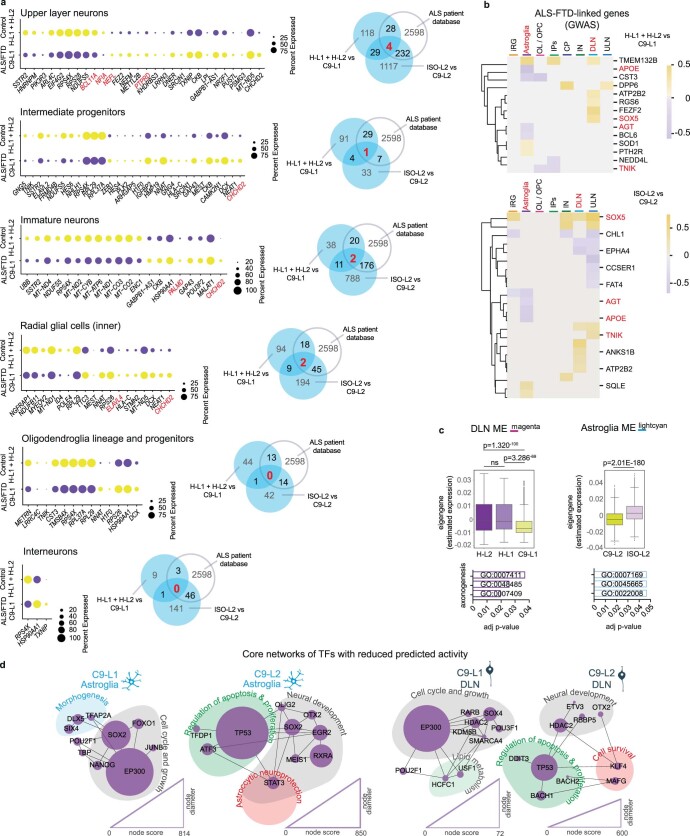
C9 ALS/FTD ALI-COs and patient samples show transcriptomic overlaps. **a**, Dot plots represent the overlap of transcriptomic changes per cell-type between C9 ALI-COs at 150 DIV and human ALS patient-related samples^20–25^. Dot size indicates the percentage of cells expressing upregulated (yellow) and downregulated (purple) genes. Venn diagrams display the number of overlapping genes between C9-L1 and/or mutation-specific C9-L2 ALI-CO DEGs (in red) and ALS-related transcriptomic changes **b**, Heatmap shows log-fold expression changes per cell-type for significant C9-L1 (upper panel) or mutation-specific C9-L2 (lower panel) DEGs overlapping with each other (in red) or with ALS/FTD-related genes taken from the ALSoD GWAS database (https://alsod.ac.uk). **c**, Boxes, lines, whiskers display the quartile, median, min-max distribution (without the outliers) representing highly correlated genes defined by top differentially expressed module eigengenes (WGCNA) for astroglia and deep layer neurons (DLN) for 2 independent ALI-CO slice-pairs per group (4 ALI-CO slices per line). Two-sided Mann-Whitney-Wilcoxon test with Bonferroni correction. Bar graphs show corresponding top significantly enriched GO terms. **d**, Core interaction networks (STRING) of transcription factors (TFs) with predicted reduced activity (SCENIC), defined by the top 10 betweenness centrality score (Cytohubba/Cytoscape) for interacting TFs (nodes) and grouped into functional domains based on protein function (www.uniprot.org). Node size illustrates centrality score. See Supplementary Table 3 for detailed statistics.

To explore cell-type-specific and disease-related changes in C9 ALI-COs, we examined differentially expressed genes (DEGs) in the scRNA-seq dataset for each cluster between the C9-L1 and H-L1/H-L2 control ALI-COs. Astroglia and DLNs displayed the highest amount of DEGs, 179 and 316, respectively (Fig. 3a,b, Extended Data Fig. 3a, Supplementary Fig. 3a and Supplementary Data 1 and 2). These cell phenotypes also featured the highest number of gene expression changes overlapping with C9 ALS/FTD-related transcriptomic data^20–25^ (Fig. 3b–d and Extended Data Fig. 3a) and ALS/FTD patient-related genome-wide association study (GWAS) hits (https://alsod.ac.uk; Extended Data Fig. 3b). To assess whether the prime involvement of astroglia and DLNs was also mutation-specific, we directly compared cell-type-related DEGs between C9-L2 ALI-COs and its genetically corrected ISO-L2 ALI-CO pair. This confirmed the major degree of transcriptomic alterations and overlaps with GWAS hits for astroglia and DLNs, while also indicating marked changes in ULNs and immature neurons (Fig. 3a, Extended Data Fig. 3b, Supplementary Fig. 3a and Supplementary Data 1 and 2). However, the proportion of those gene expression changes in C9 ALI-COs, which were also mutation-specific and overlapped with transcriptomic profiles from patients with ALS, were found to be the highest in astroglia and DLNs: 4.4% (8 out of 179 genes) and 4.3% (15 out of 316 genes), respectively (Fig. 3b–d, Extended Data Fig. 3a and Supplementary Fig. 3b). Among these, the astrocytic *C1QL1* encodes a subunit of complement C1Q (ref. ^26^), and neuronal *NRTK2* encodes the TRKB receptor, both of which are implicated in ALS pathogenesis^27^ (Supplementary Data 1 and 2). However, it is unclear how most of these genes contribute to pathology in accord. Thus, to indicate a broader astroglia and DLN-relevant pathological pathway representation, we initially performed a Gene Ontology (GO) term analysis. The most significantly enriched C9 ALI-CO-related terms and their overlaps with mutation-specific ontologies all reflected changes in protein targeting and neuronal development in astroglia and DLNs, respectively (Fig. 3e and Supplementary Data 3). These cell-type-related findings in C9 ALI-COs had been reinforced by weighted gene co-expression network analysis (WGCNA) that compensates for dropout-related bias^28^, while also suggesting changes in astrocytic extracellular matrix remodeling and synaptic plasticity (Fig. 3f, Extended Data Fig. 3c, Supplementary Fig. 3c,d and Supplementary Data 4). To help understand the functional relevance, we performed a transcription factor (TF) activity inference analysis^29^ (Fig. 3g, Extended Data Fig. 3d and Supplementary Data 5). Mapping the putative active TFs on STRING gene and protein-interaction networks, topological studies in Cytoscape identified three major functional domains within the core network of genes defined by their connectivity (betweenness) score relating to their central role in disease pathways. Among these, *DDIT3*, *SP1*, *NFE2L2* and *EGR1* in astroglia and *DDIT3*, *FOSL1*, *ATF3*, *LEF1* and *NFATC1* in neurons indicated endoplasmic reticulum (ER) stress, oxidative stress, unfolded protein response activation and DNA damage as the key altered functional domains in C9 ALI-COs (Fig. 3g). To a lesser extent, well-connected TFs also reflected astrocyte reactivity or neuronal inflammatory and developmental changes. In particular, highly connected network genes *RELA*, *NFATC1*, *FOXO1* and *CREB1* suggested activated cell death, inflammatory pathways and synaptic plasticity-related changes in C9-L2 ALI-CO versus ISO-L2 ALI-CO DLNs, which emphasizes mutation-driven changes (Fig. 3g). Core networks of TFs with reduced activity represented disturbances in lipid metabolism and generic pathways in cell cycle and growth (Extended Data Fig. 3d). However, overall, our findings predicted ER stress and DNA damage response (DDR) as the main affected pathways in astroglia and neurons in C9 ALI-COs.

### Astroglia are affected by early ER stress in C9 ALI-COs

**Fig. 4:**
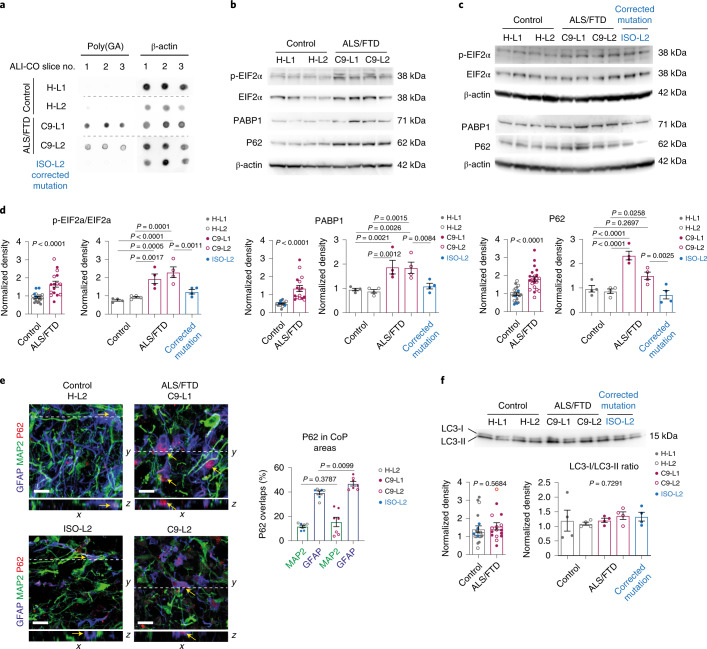
C9 ALI-COs show ALS/FTD-related pathological hallmarks at 150 DIV. **a**, Dot blot shows anti-poly(GA) immunoreactivity in C9 ALI-COs (*n* = 3 independent ALI-COs per group). **b**,**c**, Representative WB images for the ER stress/UPR elements p-EIF2α/EIF2α, the stress granule marker PABP1, the autophagy marker P62, and β-actin in ALI-CO samples from experiments quantified in **d**. **d**, Quantification of WB band densities normalized to β-actin. Left graphs: data represent the mean ± s.e.m.; for p-EIF2α/EIF2α ratios and PABP1 *n* = 20 control (H-L1, H-L2, ISO-L2) and *n* = 16 C9 (C9-L1, C9-L2) and for P62 *n* = 23 control (H-L1, H-L2, ISO-L2), *n* = 22 C9 (C9-L1, C9-L2) ALI-COs, respectively; two-tailed unpaired *t*-test. Right graphs: data represent the mean ± s.e.m. from experiments comparing C9-L2 to its isogenic mutation-corrected ISO-L2 control and non-related ALI-COs; *n* = 4 independent ALI-COs per group; one-way ANOVA with Fisher’s LSD test. **e**, Representative confocal microscopy image stacks (left) and quantification (right) of ALI-COs with single plane *x*–*z* orthogonal views, showing P62 immunolabeling in GFAP^+^ astroglia and MAP2^+^ neurons with DAPI^+^ nuclei and quantification of P62^+^ area overlaps with either GFAP^+^ or MAP^+^ territories. Data are expressed as the mean percentage ± s.e.m.; *n* = 7 independent ALI-COs per group; two-tailed unpaired *t*-test. **f**, WB image (upper) of ALI-COs, corresponding to samples in **c**, showing autophagy marker LC3 immunolabeling with bands representing LC3-I early, LC3-II late autophagosomes and β-actin. Lower left: data in the graph represent the mean ± s.e.m. of LC3-II/LC3-I ratios; *n* = 20 control (H-L1, H-L2, ISO-L2), *n* = 16 C9 (C9-L1, C9-L2) independent ALI-COs per group. Lower right: data in the graph represent the mean ± s.e.m. from experiments comparing C9-L2 to ISO-L2 control and non-related ALI-COs; *n* = 4 independent ALI-COs; one-way ANOVA with Fisher’s LSD test. Scale bar, 10 μm (**e**). See Supplementary Table 3 for detailed statistics and source data for unprocessed WB images. Source data

**Extended Data Fig. 4:**
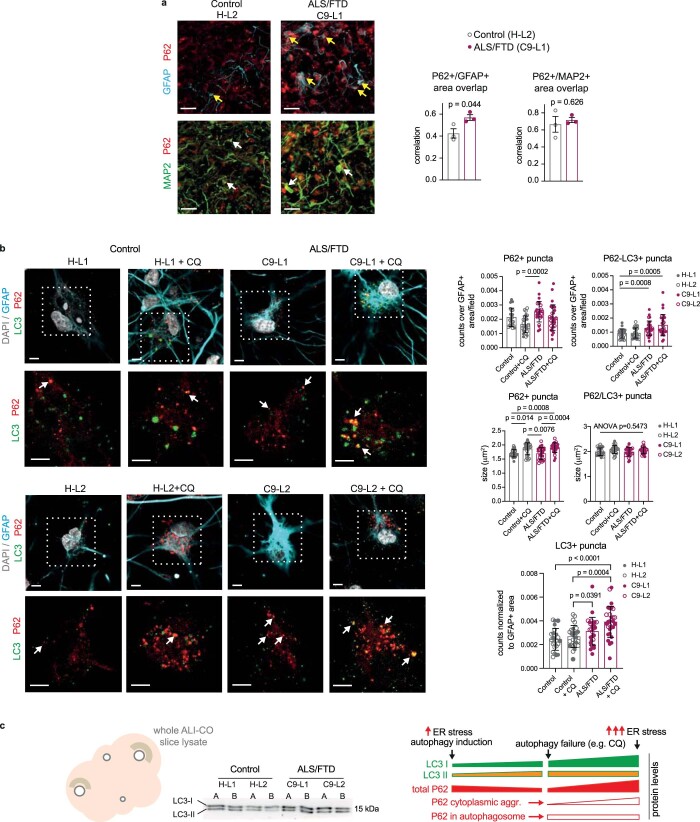
Autophagy is activated in C9 ALI-COs and astroglia. **a**, Representative whole-section confocal microscopy slide-scanner images of control and C9 ALI-COs at 150 DIV, displaying autophagy marker P62^+^ (red), astroglial marker GFAP (green) and mature neuronal marker MAP2 (cyan) labeling. Quantification of p62^+^ area overlaps with either GFAP^+^ or MAP^+^ territories, expressed as mean ± s.e.m for correlation coefficients (CellProfiler); n = 3 independent ALI-COs per group; Two-tailed unpaired t-test. **b**, Representative confocal microscopy images of dissociated control/C9 ALI-CO cell cultures (±chloroquine [CQ] treatment) immunlabeled for P62/LC3 autophagy markers. Quantification of P62^+^ and LC3^+^ punctae in GFAP^+^ astroglia. Data expressed as mean ± s.d.; n = 25, 26, 28, 29 fields (4 cultures from 6 independent ALI-COs per group); Two-way ANOVA with Tukey’s posthoc test. **c**, Western blot (WB) of whole ALI-CO slice samples, showing autophagy marker LC3-immunolabelling with bands representing LC3-I early and LC3-II late autophagosomes. Schematic (right side) illustrates typical P62, LC3 level changes in authophagy activation vs failure. Scale bar=80 μm for a, 5 μm for b. See Supplementary Table 3 for detailed statistics and Source Data for unprocessed WB images. Source data

To confirm the computational predictions, we first examined the overall activation of ER stress responses and then astroglia- and neuron-specific alterations. Since the production of DPRs by aberrant transcripts is a major trigger for ER stress in C9 ALS/FTD^30,31^, we first validated the presence of poly(GA), which is one of the toxic DPRs detected in the frontal cortex of patients^32,33^. Using an immuno dot blot assay, we detected an abundant signal for poly(GA) in whole lysates of C9 ALS/FTD organoids at 150 DIV but not in controls, including the genetically corrected ISO-L2 ALI-COs (Fig. 4a). Next, quantified western blots (WBs) were used to mark unfolded protein response (UPR) activation. This revealed a significant 1.79-fold increase in the ratio of phospho-EIF2α/EIF2α protein levels compared to controls (Fig. 4b–d), a result previously reported for C9 ALS/FTD brain samples^34^. This was accompanied by a 2.67-fold increase in PABP1 levels, a stress granule marker^35^, and a 1.86-fold increase in the total P62 protein content, an autophagy signaling protein and also a broad indicator of ER stress in C9 ALS/FTD^36^ (Fig. 4b–d). The levels of all these stress markers were significantly reduced to baseline in genetically corrected ISO-L2 organoids (Fig. 4b–d and Supplementary Fig. 4a). Then, we examined the distribution of P62 content between astrocytes and neurons to elucidate potential cell-type-specific proteostasis derangements in C9 ALI-COs. In the cortical plate areas, analysis by confocal microscopy revealed a predominant GFAP^+^ astroglial distribution of P62 immunoreactivity, which was overall increased in C9 ALI-COs compared to the controls (Fig. 4e). This was corroborated by image analysis of whole ALI-CO sections, which demonstrated a 1.25-fold higher correlation of P62^+^ areas with GFAP^+^ astroglial than MAP2^+^ neuronal territories (Extended Data Fig. 4a and Supplementary Fig. 4b). Since the overall increase in P62 levels alone would not distinguish between increased early synthesis or late autophagy clearance failure^37^, we explored its cellular distribution by high-resolution intracellular analysis in cell cultures of dissociated ALI-COs. We found a 1.6-fold greater density of P62^+^ objects overlapping with particles labeled for the autophagy marker LC3 in C9 versus control astroglia (Extended Data Fig. 4b). This suggested some degree of active autophagy, which was also supported by an unchanged proportion of early and late autophagosomes defined by LC3-I and LC3-II, respectively, protein levels detected in WBs (Fig. 4f and Extended Data Fig. 4c). To further probe this, we blocked autophagic flux using chloroquine^38^, which in pre-existing autophagy failure is expected to result in unchanged cytosolic/autophagosome P62 distribution. We found an overall increase in P62^+^ puncta size, while the density or size of individual or LC3 co-labeled P62^+^ objects and total LC3^+^ particle density did not change in either group, thereby indicating an increase in cytoplasmic P62 aggregation (Extended Data Fig. 4b and Supplementary Fig. 4c). Although further studies are required, these findings support that protein elimination pathways do not completely fail in astroglia derived from C9 ALI-COs and indicate initial proteostasis disturbances.

### DLNs are prone to DNA damage in C9 ALI-COs

**Fig. 5:**
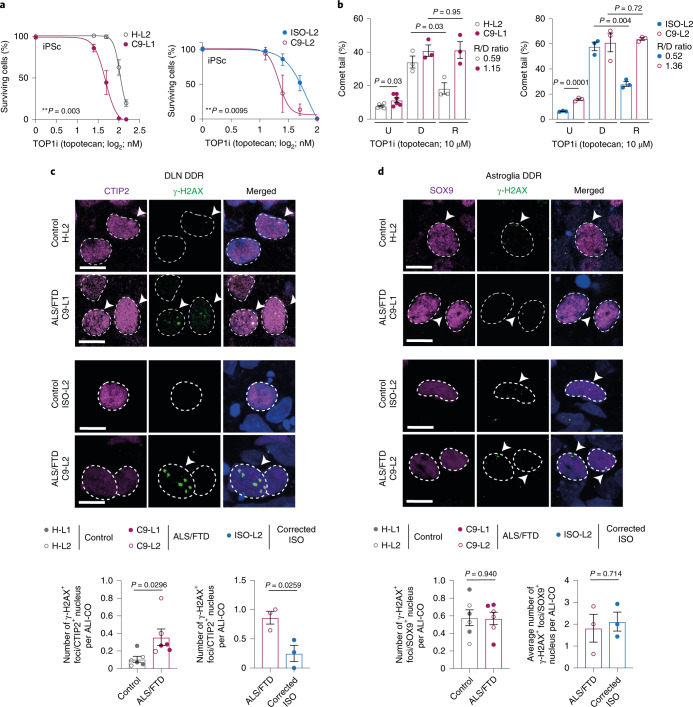
DNA damage accumulation in C9 hiPSCs and ALI-COs. **a**, Control and C9 hiPSC survival curves in response to topotecan, a TOP1i, in cultures of healthy control H-L2/C9-L1 hiPSC pairs (left) and genetically corrected isogenic control ISO-L2/C9-L2 hiPSC pairs (right). Data represent the percent of surviving cells as the mean ± s.e.m.; *n* = 3 independent cultures for each line; extra sum of square *F*-test. **b**, Quantification of comet assays for corresponding control and C9 hiPSCs (as seen for **a**) in response to TOP1i treatment. Graph represents the percentage of comet tails for untreated hiPSCs (U) and 1 h after TOP1i-induced DNA damage (D) or 1 h after TOP1i removal (R). Data show the mean ± s.e.m.; *n* = 3 independent cultures per group (for untreated H-L2, C9-L1 cells, *n* = 6 independent cultures per group; >30 cells for each). Two-tailed unpaired *t*-test. **c**,**d**, Confocal microscopy immunofluorescence images (upper) displaying γ-H2AX^+^ foci (green) in the nuclei of CTIP2^+^ DLNs (**c**) and SOX9^+^ astroglial cells (**d**) in control and C9 ALI-COs at 150 DIV and quantification (lower). Dashed circles illustrate nuclei defined by DAPI staining (blue in the merged images); γ-H2AX^+^nuclei are labelled by arrowheads. Corresponding bar plot graphs represent γ-H2AX^+^ foci for DLNs (**c**) and astroglia (**d**). Data expressed as the mean ± s.e.m. foci per cell per ALI-CO slice; *n* = 6 (left graphs) and *n* = 3 independent ALI-COs (right graphs) in 3 experiments; two-tailed unpaired *t*-test. Scale bars, 10 μm (**c**,**d**). See Supplementary Table 3 for detailed statistics.

**Extended Data Fig. 5:**
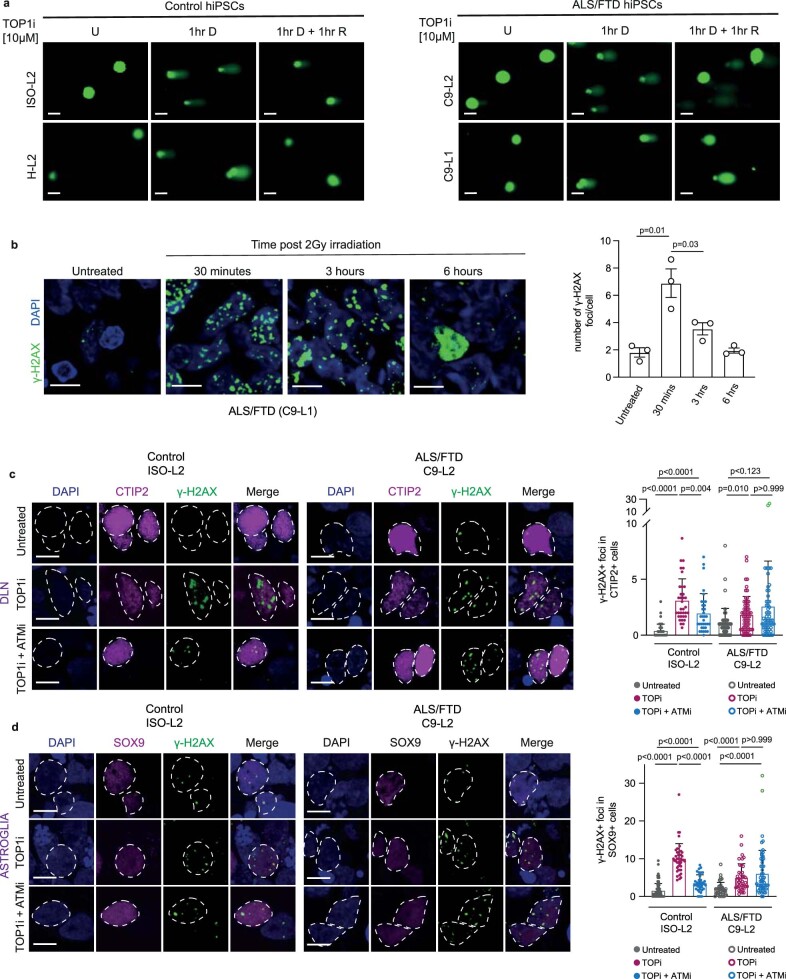
C9 ALI-CO neurons show increased genomic instability. **a**, Representative images of alkaline comet assay, showing SYBR green stained nuclei of untreated (U) control and C9 hiPSCs or 1 hour (hr) after TOP1i-induced DNA damage (D; Damage) or 1 hr after TOP1i removal (R; Recovery) from 3 independent experiments. **b**, Confocal microscopy immunofluorescence images displaying DNA damage marker γ-H2AX + foci (green) accumulation in C9 ALI-COs at 150 DIV following 2 Gy gamma irradiation (IR) and quantification. Data expressed as mean ± s.d.; n = 3 ALI-CO sections (> 120 cells for each); Two-tailed unpaired t-test. **c** and **d**, Representative confocal immunofluorescence images of γ-H2AX^+^ foci in CTIP2^+^ DLN (c) and SOX9^+^ astroglial nuclei (d) in C9-L2 vs ISO-L2 ALI-COs treated with TOP1i alone ± ATM kinase inhibitor, AZD0156 (ATMi). Quantification of γ-H2AX^+^ foci. Data expressed as individual foci/cell, mean ± s.d.; n = 95-152 DLNs (c) and n = 99-193 astroglia (d) from 3 independent ALI-COs per group. Kruskal-Wallis test with Dunn’s multiple comparisons test. Scale bar=5 µm for all images. See Supplementary Table 3 for detailed statistics.

The accumulation of DNA damage observed in samples from patients with C9 ALS/FTD^39^ poses an additional risk for cell vulnerability triggered by P62 accumulation^40^ and DPRs, including poly(GA)^41^. To determine whether acquired genetic instability is present in developmental stages, we examined the DDR in control and C9 ALS/FTD patient-specific iPSCs and in their organoid derivatives at 150 DIV. For this, we used cell survival and comet assays together with DNA-damage biomarker detection, while inducing neurodegenerative-disease-relevant cell stress by topotecan, which is a topoisomerase 1 inhibitor (TOP1i) that causes single- and double-strand DNA breaks^42^. Both C9 ALS/FTD lines were vulnerable to the TOP1i compared to the corresponding controls that included the genetically corrected C9-L2 isogenic hiPSC line (ISO-L2), thereby indicating a *C9ORF72* mutation-specific susceptibility to genetic instability (Fig. 5a). The comet assay confirmed increased DNA damage accumulation in C9 hiPSCs and indicated a DNA repair defect, whereby longer comet tails occurred in the recovery phase (Fig. 5b and Extended Data Fig. 5a). Since DNA repair failure in C9 ALS/FTD is linked with ataxia-telangiectasia mutated protein (ATM)-dependent pathways^40^, we also examined this pathway in our models. We treated control and C9 hiPSCs and ALI-COs with the TOP1i in the presence and absence of a specific ATM inhibitor (ATMi; AZD2553) and immunolabeled cells for γ-H2AX, which is an established marker for DNA damage signaling^43^ (Extended Data Fig. 5b). In contrast to astroglia, C9 ALI-CO neurons had 3.36-fold or 3.45-fold greater basal γ-Η2ΑΧ labeling than their healthy control or genetically corrected counterparts, respectively (Fig. 5c,d), which may indicate a higher DNA repair threshold. However, the TOP1i induced significant DNA damage in both cell types in addition to hiPSCs (Extended Data Fig. 5c,d and Supplementary Fig. 5a–e). The impaired rescue following ATMi administration compared to controls indicated a failure in DNA-break recognition due to ATM-signaling deficiency in all these cell types (Extended Data Fig. 5c,d and Supplementary Fig. 5a,b). These data confirm that our human ALI-CO disease model recapitulates the genetic instability previously reported in samples from patients with ALS/FTD^40^ while revealing an increased genetic risk in stem cells and in somatic post-mitotic cells, especially in neurons.

### Pharmacological rescue of cell vulnerability in C9 CO slices

**Fig. 6:**
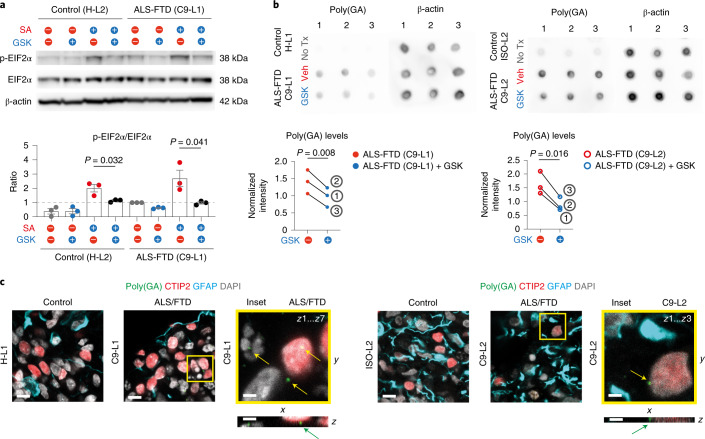
GSK reduces UPR activation and poly(GA) levels in C9 CO slices. **a**, WB image of CO slice samples at 220 DIV after immersion in medium for 12 h of GSK treatment with or without SA for 2 h. WB bands represent p-EIF2α, EIF2α and β-actin protein levels (upper) with quantification of p-EIF2α/EIF2α band density ratios after normalization to β-actin and to untreated C9-L1 controls in each blot (lower). Data are expressed as the mean ± s.e.m.; *n* = 3 independent CO slices; two-tailed unpaired *t*-test. **b**, Upper: dot blots show anti-poly(GA) immunoreactivity in C9-L1 versus H-L1 CO slice samples at 240 DIV and C9-L2 versus ISO-L2 CO slice samples at 200 DIV with or without (No Tx) a 14-day-long GSK or vehicle (Veh) treatment. Lower: scatter plots represent individual values of normalized blot densities to β-actin; *n* = 3 control–treatment CO slice-pairs from 3 independent organoids; two-tailed paired *t*-test. **c**, Representative immunofluorescence confocal superimposed *z*-stack images showing CTIP2^+^ neuronal nuclei (red) with perinuclear poly(GA) foci (green; arrows) and GFAP^+^ astroglia (cyan) overlapped with DAPI staining for three biological repeats per group. Orthogonal views (*x*–*z*) represent single confocal reconstructed planes. Scale bars, 10 μm for all images (2 μm for insets). See Supplementary Table 3 for detailed statistics and source data for unprocessed WB images. Source data

**Fig. 7:**
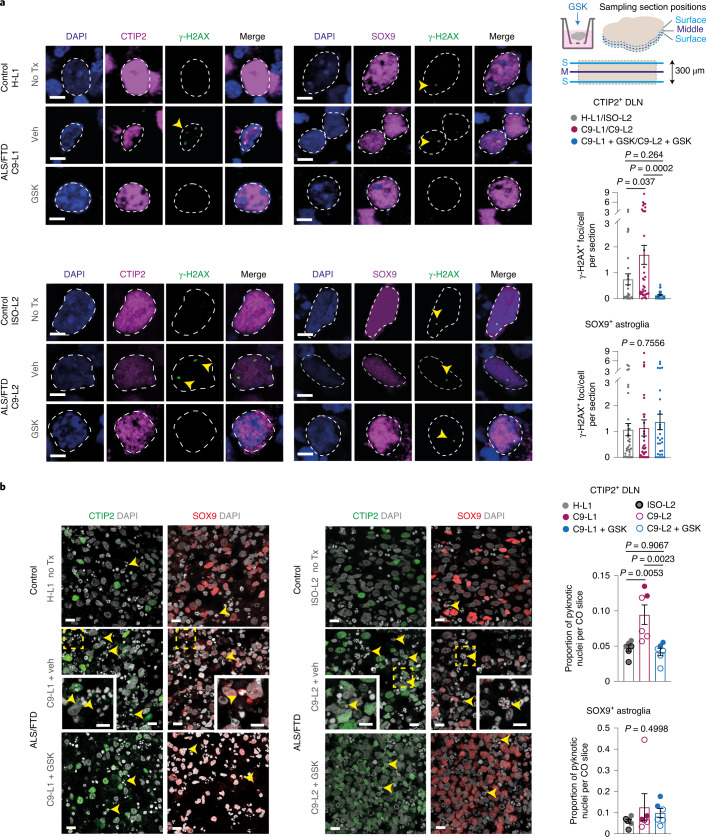
Neuronal vulnerability is distinct from astroglial stress and is partially rescuable in C9 CO slices. **a**, Left: representative confocal microscopy images showing γ-H2AX^+^ foci (arrowheads) in CTIP^+^ DLNs and SOX9^+^ astroglial nuclei in C9-L1 (240 DIV) and in C9-L2 CO slices (200 DIV) immersed in medium and treated with GSK or vehicle in comparison to age-matched untreated H-L1 or ISO-L2 control CO slices, respectively. Right: schematic (upper) of the section sampling across the vertical axis of CO slices for quantification (lower). Data represent foci numbers per cell and expressed as the mean ± s.e.m. of section averages; for DLNs, *n* = 28, 36, 30 sections from 7 control, 8 C9 and 6 GSK-treated C9 independent CO slices, respectively (including 6 independent untreated–treated C9 CO slice-pairs); for astroglia *n* = 33, 36, 29 from 8 control, 8 C9 and 6 GSK treated C9 independent CO slices, respectively (including 6 independent untreated–treated C9 CO slice-pairs); one-way ANOVA and Tukey’s post hoc test (for astroglia, overall ANOVA *P* value is indicated in the graph). **b**, Immunofluorescence confocal stack images (left) displaying pyknosis (arrowheads) in CTIP2^+^ DLNs and SOX9^+^ astroglial nuclei in C9-L1 (240 DIV) and in C9-L2 CO slices (200 DIV) treated with GSK or vehicle in comparison to age-matched untreated H-L1 or ISO-L2 control CO slices, respectively, and quantification (right). Data represent the proportion of pyknotic nuclei with residual CTIP2 or SOX9 immunoreactivity and expressed as the mean ± s.e.m.; *n* = 6 for independent control slices, and 6 independent untreated and GSK-treated C9 CO slice-pairs; one-way ANOVA and Tukey’s post hoc test (for astroglia, overall *P* value is indicated in the graph). Scale bars, 5 μm (**a**,**b** (inset)) or 10 μm (**b**). See Supplementary Table 3 for detailed statistics.

**Extended Data Fig. 6:**
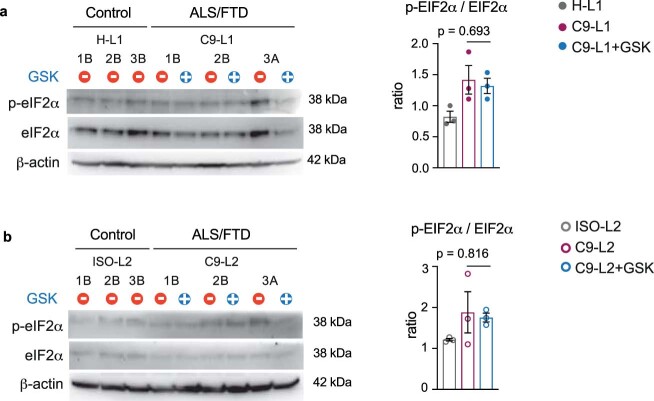
Long-term GSK treatment of fully immersed CO slices in media no longer prevents EIF2α phosphorylation. **a** and **b**, Western blots (WB) displaying p-EIF2α, EIF2α, β-actin levels in samples of H-L1 control and C9-L1 CO slices at 240 DIV (a) and ISO-L2 and C9-L2 CO slices at 200 DIV (b) immersed in media with or without 14-day treatment with GSK. Quantification of p-eIF2α and eIF2α protein levels as indication of UPR activation for H-L1/C9-L1 CO slice samples (a) and for the ISO-L2/C9-L2 CO slice samples (b). Data represent mean ± s.e.m. for ratios of band densities; n = 3 independent control CO slices and independent untreated/treated C9 CO slice pairs; Two-tailed unpaired t-test for the GSK effect. See Supplementary Table 3 for detailed statistics and Source Data for unprocessed WB images. Source data

**Extended Data Fig. 7:**
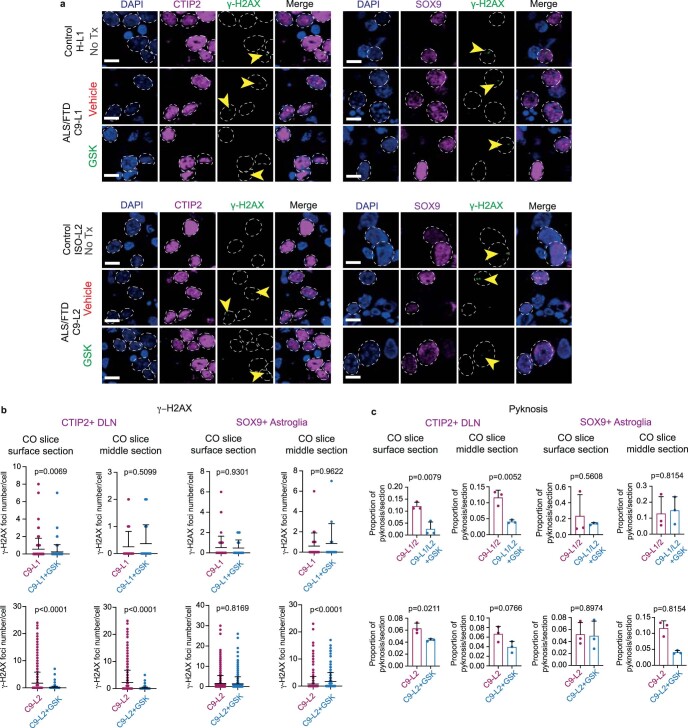
The rescue of neuronal vulnerability by GSK is more effective overall in the surface of C9 CO slices. **a**, Representative low magnification immunofluorescence images of γ-H2AX^+^ foci in CTIP2^+^ deep layer neuronal (DLN) and SOX9^+^ astroglial nuclei in C9 CO slices immersed in media and treated with GSK or vehicle in comparison to untreated controls for experiments quantified in b (8 independent untreated and 6 treated COs). Arrowheads illustrate nuclei (dashed circles defined by DAPI staining) with γ-H2AX^+^ foci (green). **b**, For C9-L1 CO slices (240DIV), graphs represent foci/cell ± s.d.; n = 105, 110 and 32, 32 CTIP2^+^ DLNs and 57, 23 and 25, 14 SOX9^+^ astroglia in surface and middle sections for C9 and GSK-treated C9 independent CO slice-pairs, respectively. For C9-L2 COs (200DIV), graphs represent foci/cell ± s.d.; n = 640, 534 and 404, 286 CTIP2^+^ DLNs and 631, 558 and 411, 274 SOX9^+^ astroglia in surface and middle sections for untreated and GSK-treated independent C9 CO slice-pairs, respectively; Two-sided Mann Whitney test for the GSK effect. **c**, Quantification of pyknosis in CTIP2^+^ DLN and SOX9^+^ astroglial nuclei in C9 CO slices treated with GSK or vehicle and in untreated controls. Data represent the proportion of pyknotic nuclei with residual CTIP2- or SOX9-immunoreactvity and expressed as mean ± s.d. for surface or middle sections; n = 3 (2 C9-L1/1 C9-L2) untreated and 3 GSK-treated independent C9 CO slice-pairs at 240 DIV (upper panel) or 3 C9-L2 untreated and 3 GSK-treated independent CO slice-pairs at 200DIV (lower panel) per group; Two-tailed unpaired t-test for the GSK effect. Scale bars=10 μm. See Supplementary Table 3 for detailed statistics.

We then explored whether the cellular vulnerability seen in C9 ALI-COs could be pharmacologically ameliorated. First, we assessed whether C9 COs at 220 DIV are more prone to UPR activation by an ALS/FTD-relevant oxidative stressor, sodium arsenite (SA), and whether overactivation can be prevented by short- or long-term treatments with GSK2606414 (GSK onwards), which is a repressor of translational inhibition caused by UPR. When ALI-COs were fully immersed in medium plus GSK solutions (CO slices), 12 h of GSK treatment reduced the ratio of total p-EIF2α/EIF2α protein levels in the C9-L1 CO slices even in the presence of SA in both controls and C9 samples as detected by WBs (Fig. 6a). Informed by the positive effect by initial short-term studies, we then extended our analysis to both C9-L1 (240 DIV) and C9-L2 (200 DIV) CO slices for long-term treatments. GSK administration for 14 days no longer had an effect on the ratio of p-EIF2α/EIF2α levels. This suggested that long-term immersion of CO slices worsened cell stress in general, which is possibly due to a decline in oxygen diffusion into the tissue^12^, thereby masking subtle GSK-induced changes or exaggerating cell toxicity^44^ (Extended Data Fig. 6a,b). However, given that GSK can also attenuate cell stress by other routes^45^, including the suppression of toxic cellular DPR content^46^, we measured poly(GA) levels by immuno dot blots in the 14-day-long treatment group. We found an overall 31% and 46% GSK-induced reduction in poly(GA) levels in C9-L1 and C9-L2 CO slices, respectively (Fig. 6b). Consistent with previous findings^32^, we detected poly(GA) only in neurons and not in astrocytes (Fig. 6c). Since poly(GA) can induce DNA damage by impeding the function or availability of the DNA repair proteins ATM and HR23, which are key proteins involved in DNA repair^41^, we next addressed whether neuronal DNA damage can be rescued by long-term GSK treatment. We found that γ-H2AX foci accumulation in DLN populations was significantly reduced by GSK in C9 CO slices (Fig. 7a), which overall was more prominent in the surface sections of C9 slices (Extended Data Fig. 7a,b). In contrast, no significant differences were detected in these responses in astrocytes in C9 COs (Fig. 7a and Extended Data Fig. 7a,b). This suggests that there is an increased susceptibility of neurons associated with detectable poly(GA) levels compared to astrocytes lacking poly(GA). Finally, to validate this selective neuronal susceptibility in C9 CO slices, we measured nuclear pyknosis of CTIP2^+^ DLNs and SOX9^+^ astrocytes. Overall, C9 CO neurons but not astrocytes showed significantly increased pyknosis compared to controls. This could be partially suppressed by GSK (Fig. 7b), especially in the surface regions of C9-L1 and C9-L2 organoid slices (Extended Data Fig. 7c). Altogether, our results suggest cell-type-specific and targetable cell vulnerabilities in C9 cortical organoid slices.

## Discussion

We reported the development of a novel 3D human cortical ALS/FTD organoid slice culture model that reliably recapitulated mature astroglial and neuronal phenotypes and displayed distinct molecular pathological hallmarks. Transcriptomic signatures at single-cell resolution reflected disturbances in cell homeostasis, which, in combination with biological validations, demonstrated cell-subtype-selective susceptibilities, an early predominant ER stress response in astrocytes and a pharmacologically reversible increase in poly(GA) levels, DNA damage and cell death in deep layer cortical neurons.

Capturing risks and disease-causing pathology in somatic cells, which are already present in the preclinical stages in hereditary or sporadic neurodegenerative diseases, has been challenging^4^. This can be due to the relative inaccessibility of samples from presymptomatic patients or the lack of dynamic model systems that can reliably mimic the complexity of human neuropathology. This work also supported recent suggestions that organoid slice-culture preparations are robust in representing cell-subtype maturity^5,6^, thereby highlighting important improvements in standardizing methods for human disease modeling. Although previous animal and stem-cell-based models have implicated many cell types in ALS and FTD pathogenesis^13^, human cell models have been limited in resolving the temporal relationship of early cell-type-specific changes. The C9 ALI-CO slice culture system is optimal for such investigations due to the long-term multi-cell cohabitation of consistently formed cortical domains.

Our scRNA-seq studies confirmed the two major cell types^13^ involved in ALS/FTD pathology while also providing clues on initial astroglia- and DLN-specific pathway disturbances that reflect the broad themes in ALS/FTD pathology. These included disruptions in protein homeostasis, DNA repair, reactive astrocyte response, inflammatory activation and neural development. It is notable that the latter concerns neuronal projection morphogenesis and synaptic plasticity pathways, which emerged as consistent hits in recent ALS/FTD-related^47^ and other neurodegenerative-disease-related^9^ studies. We speculate that in our paradigm, these terms can also represent adaptive neuronal plasticity given that synapse density and network activity in C9 ALI-COs remained unchanged compared to controls at 150 DIV, despite a modest increase in cell loss. This is supported by recent studies providing evidence for synaptic plasticity at early disease stages in animal ALS or ALS/FTD models before its failure^48,49^. Long-term ALI-CO cultures could now provide an opportunity to explore the timing and causative relationship between synapse and neuronal loss, which could not be mechanistically examined before in a human disease context.

Analysis of mutation-specific changes and their overlap with ALS transcriptomic datasets increased the confidence in the model platform. Similarities and differences between cell-line-dependent or mutation-dependent overlaps in gene expression changes and their representation in samples from patients with ALS/FTD question the degree to which individual expression traits versus mutations underlie pathological pathways in astrocytes and DLNs. Recent large-cohort genetic studies have begun to address polygenic influences on mutation-driven ALS pathology in different cell types^47^. However, further genetic analyses and verification will be required using human biological assays. As an initial step, our paradigm demonstrates that ALI-COs can probe cell-type-specific susceptibility and vulnerability in ALS/FTD.

In this work, biological assays validated the mutation-specific contribution to UPR activation, stress granule and P62 autophagy marker increases. Although the early astroglia-specific increase of P62 content in C9 ALI-COs was modest, together with its greater immunoreactivity in astroglia versus neurons, it indicated distinct proteostasis disturbances. The unaltered ratio of LC3-II and LC3-I, markers of late and early autophagosomes, respectively, suggested active autophagy without failure, thereby highlighting ER-stress-related adaptive mechanisms in astroglia. Other compensatory events in C9 ALI-CO astroglia include a potentially higher DNA repair threshold, as indicated by a greater number of basal γ-H2AX^+^ foci without an increase in pyknosis when compared to neurons. This highlights their distinct resilience over DLNs, which had a higher pyknosis rate in C9 ALI-COs versus controls. One plausible explanation for this difference is the lack of the toxic DPR poly(GA) in astrocytes and its *C9ORF72* mutation-dependent presence in DLNs. This corresponds to observations in post-mortem brain samples from patients with ALS/FTD^32^. Poly(GA) can directly contribute to neuronal death by worsening proteostasis^33^ and impeding DNA repair proteins, such as ATM^41^. Since our study suggested that ATM signaling deficiency underlies the DNA repair failure in DLNs, and is supported by recent analyses of human cell lines^40^, poly(GA) is a prime candidate contributing to excessive DNA damage in C9 ALI-CO DLNs. This is aligned with the parallel reduction in poly(GA) levels, γ-H2AX foci and pyknosis in C9 ALI-COs by GSK, which has demonstrated effects on DPR content^45,46^ irrespective of its action on the UPR. This is highly relevant to the disturbances observed in deep layer corticospinal neurons contributing to motor dysfunction in ALS/FTD and supports the susceptibility of the primary motor cortex to such effects^50^. Whether the onset and characteristics of the observed changes also depend on the effect of hexanucleotide repeat length as a proposed disease modifier^51^ in C9 ALS/FTD is unclear. Although our findings suggested common changes between the two variants of C9 organoid lines, more extensive direct comparisons in the ALI-CO system may help reveal how repeat-length variations affect pathology. In summary, while our results match many findings seen in clinical samples^13^, they also revealed distinct astroglia- and DLN-specific differences in early pathological pathways.

An unresolved issue is how the initial, proteostasis-related changes in astroglia contribute to neuronal pathology in C9 ALI-COs. Despite recent evidence of neurotoxicity induced by a similar astroglial phenotype with UPR activation^52^, and the well-described contribution of astrocytes to neuropathology in ALS or ALS/FTD^13^, the functional consequences of C9 ALI-CO astrocytes have yet to be explored. This is pertinent to our organoid model as the transcriptomic data also point to secondary reactive astroglial responses, which can be associated with protective effects. Thus, teasing apart the primary and secondary astrocyte pathology and their functional consequences on neurons through perturbation experiments in C9 ALI-COs would be a therapeutically relevant goal^53^.

Despite the advances our C9 CO slice model represent, there are limitations to be addressed. In our paradigm, the organoid culture method suppresses mesoderm-derived immune cell/microglia presence^54^ to achieve a more consistent forebrain identity and cell composition. This allowed us to specifically address initial astroglia- and neuron-dependent pathologies without immune or secondary inflammatory responses. However, supplementation of ALI-COs with microglia/allogeneic immune cells could address immune-related pathogenesis in ALS/FTD in the future^55^. For instance, a recent study^56^ showed that human blood-derived macrophages and brain tissue from patients with C9 ALS/FTD show an elevated type I interferon signature, and that *C9orf72* deletion in mice leads to autoinflammation. Therefore, it would be exciting to address how immune cells affected by the *C9ORF72* mutation could contribute to cell vulnerability and its spatiotemporal spread in ALI-COs.

The partial pharmacological rescue of early protein homeostasis- and DNA damage-related pathogenesis in our patient-specific organoids illustrates how C9 CO slices can be used for personalized and broader drug discovery strategies. Sliced organoids provide an important advantage as two adjacent slices derived from the same organoid could serve as a control–treatment pair, increasing the confidence in analyzing drug-related effects. The slicing of organoids may improve nutrition, suppressing the necrotic core^5^ that often features in whole organoids. However, we cannot exclude the possibility that the complete immersion of slices in the medium for optimized treatments had decreased oxygenation, as previously seen in whole organoids^12^. Therefore, during long-term drug administration, this may suppress early and more subtle differences in stress responses between control and C9 organoid slices or the rescue effects, while not affecting robust changes, such as seen for poly(GA) levels. While immersed organoid slices are optimal for shorter term drug treatments, slices cultured at the ALI could be a preferred platform for longer term drug testing.

Scaling up this approach using many patient cell lines for more comprehensive drug screening applications would require further technical efforts and automation for handling simultaneous long-term cultures. However, the consistent cell composition between the different ALI-CO lines/batches, comparison to genetically corrected ALI-COs and datasets of patients with ALS, and validated cellular disturbances across the lines provide confidence in future mechanistic studies. Altogether, our results suggest a dormant perinatal or presymptomatic cortical vulnerability, an emerging concept in ALS and related diseases^4,19^, and open up a unique opportunity to investigate the relationship between underlying risks and the pathological phenotype^57^. In conclusion, the generation of C9 ALI-COs delivers a unique platform for the translational neuroscience community, enabling the exploration of selective cell vulnerabilities and novel personalized diagnostic and therapeutic approaches for ALS/FTD.

## Methods

### hiPSC culture

hiPSC lines were obtained from three suppliers (Supplementary Table 1). Cell lines were adapted and cultured in StemFlex medium (Thermo Fisher Scientific, A3349401) on plates coated with Geltrex (Thermo Fisher Scientific, A1413302). Cells were fed daily and passaged using 0.5 mM EDTA (Thermo Fisher Scientific, 15575020) when confluency reached 60–70%. For banking, cells were frozen in StemFlex medium containing 10% dimethylsulfoxide (Sigma Aldrich, D2650).

### Generation of cerebral organoids and ALI-COs

For the generation of hiPSC-derived COs cultured at the ALI, we modified a previously published method^5^ utilizing a STEMdiff Cerebral Organoid kit (StemCell Technologies, 08570). Briefly, 18,000 cells were plated in the presence of poly(lactide-co-glycolide) copolymer microfilaments to achieve improved cortical development^58^. Initially, media changes were performed at days 3, 5 and 7, then every 3–4 days. From 35 DIV onwards, Matrigel (Corning, 354234) was added in 1:50 dilution to achieve a polarized cortical plate formation. Between 50 and 80 DIV, 300-μm thick ALI-CO cultures were prepared for subsequent long-term cultures on Millicell-CM (Merck Millipore, PICM0RG50) inserts and were fed daily with slice medium containing neurobasal medium (ThermoFisher Scientific, 21103049) supplemented with 1× B27 supplement (ThermoFisher Scientific, 17504044), 0.45% (w/v) glucose (Sigma-Aldrich, G8769), 1× Glutamax (ThermoFisher Scientific, 35050038) and 1% antibiotic–antimycotic (ThermoFisher Scientific, 15240062).

### Organoid cell dissociation

For organoid cell dissociation, slices were transferred into a 10-cm^2^ dish containing 1× dPBS (Sigma Aldrich, D8537). Washed slices were then placed into a gentleMACS C tube (Miltenyi, 130-093-237) containing 2 ml of papain solution (20 units per ml; Worthington, PAP2) and ran on a gentleMACS Octo dissociator (Miltenyi) using the default ABDK program. After dissociation, the cell suspension was triturated, diluted with dPBS containing 0.5 mg ml^−1^ DNAse (Sigma Aldrich, 11284932001) and then spun down at 300 × *g* for 5 min. The cell pellet was resuspended and filtered through a 70-μm strainer (Miltenyi, 130-098-462) to remove any remaining aggregates before centrifugation again under the same conditions. For scRNA-seq samples, the cell pellet was resuspended in dPBS (316 cells per μl) containing 0.04% BSA (Sigma, A9418), and the suspension was kept on ice for 30 min until being processed. For the cell culture experiments, cells were resuspended in N2B27 medium containing 10 μm Y-27632 (Tocris, 1254/10) and plated on coverslips pre-coated with polyethylenimine (Sigma Aldrich, P3143) and Geltrex.

### ER stress and rescue assays

For ER stress induction, ALI-COs at 220 DIV were treated with 50 μM SA (Sigma-Aldrich, 1062771000) or vehicle (equal volume of slice medium). For short-term rescue experiments, slices were either pretreated with 5 μM GSK (R&D Systems, 5107/10) or vehicle overnight in inserts, followed by their immersion in slice medium in 24-well plates for an additional 4 h of treatment with GSK or vehicle before SA or vehicle administration. For the long-term rescue experiments, control and C9 CO slices at 200–240 DIV were kept on inserts but were immersed in medium and fed daily with either vehicle only or supplemented with 5 μM GSK for 14 days. Each untreated–treated pair of CO slices consisted of two adjacent slices derived from the same whole organoid, and at least three independent CO slice pairs (derived from different whole organoids) were used for assessing GSK-mediated effects in each experiment.

### Real-time cell vulnerability assay

Six-well plates (Appleton Woods, Corning, CC010) were coated with 1% Geltrex (Thermo Fisher Scientific, A1413302) in DMEM/F-12 (Fisher Scientific, Gibco, 11514436). hiPSCs were seeded in StemMACS iPS-Brew XF (Miltenyi Biotec, 130-104-368) medium at a density of 1 × 10^5^ cells per well. After 24 h, the survival assay was performed in IncuCyte chambers at 37 °C and 5% CO_2_, and cells were either left untreated or treated daily with 1.25 nM, 2.5 nM, 5 nM or 10 nM topotecan (Apexbio, B2296). Images were acquired every 6 h over a duration of 5 days using a ×10 magnification objective of the Incucyte S3 Live-Cell Analysis system (Sartorius, 4647). For assessing cell viability, cell confluency was determined as a cell body cluster area by phase microscopy using the IncuCyte NeuroTrack software^59^.

### Alkaline comet assay

The alkaline comet assay was performed to compare DNA accumulation and repair in control and C9 ALS/FTD hiPSC lines^60^. Cells were seeded overnight before topotecan (10 μM for 1 h) treatment in the presence or absence of an ATMi (Selleck Chemicals, AZD0156, S8375; 30 nM for 1 h). Half of the plates were trypsinized (0.25%, Sigma-Aldrich, T4049) for the assessment of DNA damage load (damage; D). The rest were washed in PBS and further incubated for 6 h in StemMACS iPS-Brew XF (Miltenyi Biotec, 130-104-368) without topotecan or ATMi before trypsinization to allow for DNA repair (recovery; R). The cells were then resuspended in 1× PBS (Mg/Ca-free) (Sigma-Aldrich, D8537) at 2 × 10^5^ cells per ml concentration. Cell suspension (75 μl) was mixed in 500 μl LMAgarose (Trevigen, 4250-050-02; melted for 5 min at 100 °C and kept at 37 °C), then 70 μl of the mixture was pipetted on preheated (37 °C) 1% agarose-coated glass slides. The cell-containing droplets were covered with a coverslip and kept in the dark for 30 min at 4 °C before the application of the CometAssay lysis solution (Trevigen, 4250-050-01) followed by the alkaline unwinding solution (200 mM NaOH, 1 mM EDTA, pH > 13) for 1 h at each step at 4 °C in the dark. The slides were subjected to electrophoresis at 35 V for 10 min in the alkaline electrophoresis solution (200 mM NaOH, 1 mM EDTA, pH > 13). Following fixation with 70% ethanol and drying at 37 °C, slides were stained with SYBR green I (Invitrogen, in 10 mM Tris-HCl, pH 7.5, 1 mM EDTA, pH 8.0). The tail percentage was measured using the OpenComet plugin for ImageJ (SRC V1 version). For each condition, the comet tail percentage was measured (>50 cells).

### Western blotting

Cell lysates for protein samples were obtained from ALI-CO slices at 150, 200, 220 and 240 DIV using RIPA lysis buffer (Sigma-Aldrich, R0278) plus protein and phosphatase inhibitors (Thermo Fisher Scientific, 31462, A32957), and standard immunoblotting protocols were used. Briefly, 18 μg of protein was resolved by SDS–PAGE then transferred onto polyvinylidenedifluoride membranes before overnight incubation with primary antibodies (Supplementary Table 2), except for the directly conjugated β-actin antibody, for which 1-h incubation was applied. Species-specific horseradish-peroxidase-conjugated secondary antibodies were applied for 1 h at room temperature at 1:10,000 dilution (Supplementary Table 2) before signal detection with the enhanced chemiluminescence system (GE Healthcare, RPN2232). Standard quantitative WB analysis was performed in ImageJ, and band density levels were expressed as fold-changes to controls after normalization to β-actin and controls within the same blot.

### Immunocytochemistry

ALI-COs and whole organoids at 30 and 75 DIV were fixed in 4% paraformaldehyde (PFA) for 45 min, 2 or 4 h, respectively, at room temperature. Fixed samples were prepared as frozen blocks for cryostat sectioning, and 12-μm thick frozen sections were immunostained with antibodies (Supplementary Table 2) using our published protocols^61^. For immunofluorescence-based detection of DDR in hiPSCs, cells were seeded on 1% Geltrex-coated coverslips 24 h before treatment (Geltrex, Thermo Fisher Scientific, A1413302). After treatment, cells were washed in PBS followed by fixation in 2% PFA (Alfa Aesar) for 15 min at room temperature, then washed again three times in PBS. hiPSCs and ALI-CO cryostat sections were then permeabilized with 0.5% Triton X-100 (Sigma-Aldrich, T8787) for 10 min, before blocking at room temperature for 1 h in 5% BSA (w/v; Sigma, A9647), followed by primary antibody (in 5% BSA PBST) incubation overnight at 4 °C. Cells were incubated with secondary antibody (in 5% BSA PBST) for 1 h at room temperature and were counterstained with 4,6-diamidino-2-phenylindole (DAPI; 0.2 mg ml^−1^) and then were mounted using Fluoromount-G (Affymetrix eBioscience, 15586276). For immunolabeling-enabled 3D imaging, organoids were solvent-cleared using the published iDISCO method^62^.

### Image acquisition and processing

Images were acquired using a confocal microscope (Leica TCS SPE, *z*-stack step: 0.5–1 μm, ×20–63 objective; 1,024 × 1,024 or 2,024 × 2,024 pixel images), an automated confocal slide scanner (Pannoramic Confocal, 3DHISTECH, 9,216 × 11,520–15,616 × 15,872 pixel ranges for images), a confocal spinning disk microscope (Andor Dragonfly 302, ×20 objective, 1,177 × 1,177 pixel images) or a fluorescence microscope (Leica DM6000, ×10 objective, 1,392 × 1,040 pixel images). Camera exposure and gain were kept the same while collecting images for each experiment. Unmodified images were used for manual analyses. For unbiased semi-automated analyses of the cell nucleus and immunoreactive SYT1^+^, HOMER^+^, P62^+^, LC3^+^ and 53BP1^+^ particle counts, automated batch normalization with background fluorescence signal subtraction was uniformly performed on images using established image analyzer software plugins with settings specified in the relevant method sections. Automation was verified by manual cell and particle counts. For illustration purposes, the recommended guidelines were followed. Representative images were only minimally and uniformly processed in ImageJ (v.2.0.0 Fiji) or in Adobe Photoshop without affecting data presentation. This included changes in exposure and/or contrast parameters (= 0.3) when clear views were obscured in merged images by interference between strong DAPI/cytoplasmic GFAP staining and other immunoreactive objects (SOX2, SOX9, SATB2, CTIP2, P62 and LC3) on coverslips and histological slides. Cyan or magenta pseudocolors were rendered to images in ImageJ for immunolabelling of SOX9^+^, CTIP2^+^ cell nuclei (magenta or gray) for multicolor visualization. For WB densitometry, chemiluminescence on WB membranes was detected by the Alliance 4.7 CCD image system (UVITEC), and the original membrane images were used. For focused illustration, the images were cropped, leaving a minimum of six band width in all lanes with corresponding β-actin loading controls. For figure assembly, images were embedded in Adobe Illustrator. For schematic illustrations, parts of drawings from the Motifolio drawing toolkit were utilized (www.motifolio.com) for figure preparation.

### Synapse density analysis

For synapse analysis of ALI-COs, *z*-stacks of images were taken from 3 cortical plate areas in 11–17 sections per each ALI-CO by confocal microscopy, using the same parameters set for the control samples (×63 lens, ×1.5 digital zoom, 1,024 × 1,024 image resolution, phase correction *x* value = −33.4). This included four to six ALI-COs for each hiPSC line. For *z*-stack images, standard batch image-normalization was performed in ImageJ by applying a 0–255 range histogram stretch (no pixel value alteration) and the “subtract background” function^63^ using the same parameters for the entire dataset. Synapse quantification was carried out using the open-source CellProfiler software^64^ (v.3.1.9; http://cellprofiler.org) with optimization of a publicly available pipeline (10.7488/ds/2132). Using the MaskObjects function, masks for presynaptic and postsynaptic particle recognition reflected close proximity between SYT1 and HOMER1, respectively (synaptic puncta size threshold = 6–15 pixels). From each *z*-stack image, 3–4 confocal planes at 1 μm apart were analyzed to prevent double counts for synapses (96–178 images per group). Synapse densities were expressed as the number of colocalizing masks over areas defined by MAP2^+^ dendrites identified by the “tubeness” function in CellProfiler.

### P62 and autophagy marker detection

For the analysis of P62^+^ and LC3^+^ particles in astroglia, ALI-CO slices at 190–220 DIV were dissociated as described above. Cells (20,000) were plated on nitric-acid-treated coverslips, coated overnight with polyethyleneimine and subsequently with Geltrex for 4 h at 37 °C. After 20 days, cultures were treated with the autophagy inhibitor chloroquine (Autophagy Assay kit, Abcam, 139484) or vehicle for 18 h at 30 μM concentration. Following immunostaining of dissociated cells, *z*-stack images were taken from 3–5 different areas per coverslip by confocal microscopy (×63 lens, ×1.5 digital zoom, 1,024 × 1,024 image resolution, phase correction *x* value = −33.4) using the same settings. Principles of *z*-stack image sampling and the settings for batch image normalization in ImageJ were the same as described for synapse analysis, and immunoreactive particles were quantified using CellProfiler. Using the MaskObjects function, particle recognition was carried out within GFAP^+^ areas. The counts and sizes of individual and overlapping P62^+^ and LC3^+^ puncta were measured for comparison between control and C9 ALI-CO-derived GFAP^+^ astroglia. For the analysis of cell-type-specific distribution of P62^+^ particles in whole ALI-CO sections, immunolabeled cryostat sections were imaged using a confocal microscope or a confocal microscopy slide scanner as described above. The colocalization pipeline was used to elucidate differences in the overlap of P62^+^ areas with GFAP^+^ astroglial/MAP2^+^ neuronal territories in control versus C9 ALI-COs. Data are expressed as the proportion of overlapping areas or as a correlation coefficient generated by CellProfiler.

### DNA damage detection

DNA damage accumulation was assessed by immunolabeling for γ-H2AX or 53BP1 (refs. ^43,65^). hiPSCs were seeded overnight on coverslips (Academy, NPC16/13) coated with 1% Geltrex (ThermoFisher Scientific, A1413302; diluted in DMEM/F-12, Fisher Scientific, Gibco, 11514436).﻿ After 1 h of treatment with 10 μM topotecan, a TOP1i, alone or in combination with 30 nM of an ATMi, AZD0156, coverslips were washed with PBS containing 0.1 % Tween-20 (PBST) and were fixed with 2% PFA (w/v; Alfa Aesar, 43368) in PBS for 10 min. ALI-CO slices were first incubated in a 24-well plate, floating in 1 ml slice medium for 2 h at 37 °C, then in 1 ml of medium alone or supplemented with 10 μM TOP1i alone or in combination with 30 nM AZD0156 for 1 h. Coverslips and ALI-CO cryostat sections were processed as described in the immunohistochemistry section. To verify the specificity of DNA damage accumulation, ALI-CO slices were exposed to γ-radiation (Xhtrahl, RS225M; 2GY), and subsequently DNA repair was allowed for 30 min up to 6 h. This also served for calibrations of the DNA damage analysis pipeline for C9 hiPSCs or in ALI-COs (Extended Data Fig. 5b). Confocal microscopy (Zeiss, LSM880 100) imaging parameters were set to the intensity of γ-H2AX immunoreactivity in a control ALI-CO or CO section, which were kept uniform, and stack images were analyzed using the CellProfiler software^66^.

### scRNA-seq pipeline and analysis

The initial scRNA-seq data-analysis pipeline was generated using the CellRanger 3.1 software package according to our published protocols^5^. Reads were aligned to the GRCh38 human genome. CellRanger detected 85% fraction reads on average per cell and 1,600–2,100 median genes per cell. Bias from false cell discovery due to potential ambient RNA contamination/low UMI counts was eliminated in two samples using the “force cell” function in CellRanger. Data were processed in Seurat 3.1/4.0.1 and filtered based on a minimum of three cells expressing each gene. As a standard method, only those cells were included in the analysis that expressed 200–5,000 genes and in which the proportion of mitochondrial genes were below 25% out of all genes. Cells displaying a greater fraction in mitochondrial transcripts are conventionally regarded as nonviable and were discarded to avoid bias in the transcriptomic analysis, resulting in a dataset representing 148,223 cells. Clustering was performed in Seurat over 14 dimensions with a resolution of 0.4, and cell type or state identities were determined by the expression of previously defined markers. For merged representation of all samples, a subset of 75,497 cells was taken for batch correction using canonical correlation analysis^67^. Cell maturity analysis was achieved by independently projecting data from ALI-COs onto two different scRNA-seq datasets for fetal brains^16,17^ using the scmap 1.12.0 software^18^ (‘scmapCluster()’ threshold = 0)). For each cell type, the proportion of cells projecting to a particular actual age of the fetal brain was plotted. For trajectory reconstruction, Monocle 3 (v.0.2.3.0) was used on the entire merged dataset. Cells with a transcriptomic signature of cell stress^11^ were removed and 75,497 cells were processed. The top 3,000 variable features calculated by Seurat over 100 dimensions were used for the ‘preprocesscds()’ function. iRG cells marked by expression of TOP2A, SOX2, VIM and NUSAP1 were used as the root for ‘order_cells()’. DEGs were obtained for each batch independently (batch 1: C9-L1 versus H-L1/H-L2; batch 2: C9-L2 versus ISO-L2) and were only retained if significantly different (adjusted *P* value < 0.05) for both individual and combined comparisons. Overlap was then identified between the batch-specific list of DEGs. WGCNA was used to reveal potentially affected pathways in each cell type in the two separate batches of ALI-CO datasets using the top 3,000 variable features (WGCNA v.1.70-3). A minimum module size of 15 and a deep split of 4 were retained. The resulting modules were then projected onto the merged dataset using ‘moduleEigengenes()’. Significant differences between module eigengene values for C9 versus controls are presented in box plots. Overlap between genes from each module and DEGs calculated for the cell type was then plotted using Python. In addition, STRING (https://string-db.org) was utilized for network interaction analysis (‘medium confidence’), and the disconnected nodes were eliminated. Finally, enrichment analysis was performed for gene sets included in the networks of highly correlated genes by GO analysis via the EnrichR platform. The –log() of adjusted *P* values or the false discovery rate was taken to generate clustermap plots. To determine whether C9 ALI-COs recapitulate gene expression changes seen for C9 ALS/FTD samples, the expression of cell-type-related DEGs were compared to upregulated and downregulated genes detected in human samples from patients with ALS/FTD using various publicly available databases^20–25^. To infer a potential functional relevance for differentially affected genes in C9 ALI-COs, TF activity analysis was performed using SCENIC v.0.9.6 (python 3.6)^29^, for which a standard pipeline was followed (GRNboost2, ‘ctx’, AUCell). The resulting matrix of cells and TFs were binarized using the recommended threshold calculation in R (v.4.0.3). For each cell type, the proportion of cells with ‘active’ TFs was calculated in C9 versus control datasets.

### Southern blotting

DNA was extracted from the hiPSC cultures using a Qiagen DNeasy Blood and Tissue kit (69506). DNA (5 µg) was restriction digested with Bsu36I (New England Biolabs, R0524S) and ran on 0.8% agarose gels accompanied by molecular weight markers II (Roche, 11218590910) and III (Roche, 11218603910). Southern blotting was performed according to published protocols^68^ using a 1-kb probe (Prepared by IDT; sequence: GGGGCC) in salmon sperm DNA (ThermoFisher Scientific, 15632011) for hybridization and an anti-DIG AP antibody (Roche, 11093274910) for visualization.

### Repeat-primed PCR

DNA was extracted from samples using a QIAamp DNA Mini kit (Qiagen, 51306), and the *C9ORF72* locus amplified by PCR with the 6-FAM-labeled forward and reverse primers (see below) as previously described^69^. The PCR product was denatured and analyzed by capillary electrophoresis on an Applied Bioscience 3730XL DNA Analyzer (Thermo), and chromatographs were aligned in GeneMapper v.6. software (Thermo).

Forward primer: MRX-F1: FAM-TGTAAAACGACGGCCAGTCAAGGAGGGAAACAACCGCAGCC;

Reverse primers: MRX-R: CAGGAAACAGCTATGACCGGGCCCGCCCCGACCACGCCCCGGCCCCGGCCCCGG,

MRX-M13R: CAGGAAACAGCTATGACC

### MEA recordings

ALI-COs were secured with a platinum harp onto 3D MEAs (Multi Channel Systems, MEA2100, 60–3DMEA200/12iR-Ti-gr, 60 electrodes, 12 μm in diameter, 200-μm spacing). Six-minute-long recordings of spontaneous activity at 37 °C (*n* = 35 ALI-COs) were acquired and exported to Matlab (MathWorks) for analysis^5^. The raw signal was bandpass-filtered (third-order Butterworth, 600–8,000 Hz) and spikes detected using a threshold of 3 standard deviations (s.d.) above background noise using a 1.5-ms refractory period after each spike. Correlated activity between electrodes was analyzed using the spike-time tiling coefficient (STTC)^70^ with a synchronicity window of 175 ms. Using graph theory, the functional connectivity is shown as the edge weights and node degree of each electrode for STTC > 0.6. The node degree distribution and binary connection matrices were compared against surrogate graphs of synthetic spike matrices for temporally randomized spike trains with equivalent spike rate distribution.

### Statistics and reproducibility

The subject identifiers were blinded for the observers. Details of statistical tests and exact sample sizes are listed in Supplementary Table 3. Briefly, the sample sizes using hiPSC lines and organoids were estimated from previously performed experiments^5,61^. In total, 233 whole COs were used for this study, and 587 ALI-CO slices were grown deriving from 99 independent whole COs that were generated from three control and two disease lines harboring the *C9ORF72* mutation. Sample allocations into groups included independent organoids, ALI-COs or immersed CO slices grown from different cell lines and/or as separate batches (independent biological replicates). ALI-COs or immersed CO slices derived from identical whole organoids were only used in separate studies or as adequate control–treatment slice pairs for each independent biological replicate per group. Studies carried out on cultures of non-differentiated hiPSCs included two disease and two control lines (one of which is a genetically corrected isogenic line) in at least three independent experiments. Experiments were repeated three times (or two times for GSK treatments), which included at least three independent biological replicates, and all had similar results. At least three independent biological replicates were used per group for all statistical analyses in biological experiments. For the anti-γ-H2Ax antibody validation studies (using irradiation; Extended Data Fig. 5b), three sections sampled from different positions within one organoid were subjected to analysis for each time point. The GraphPad software (GraphPad Prism v.7.0/8.0) was used for distribution analysis, statistical analysis and for generating graphs. When normality was not assumed or defined, nonparametric tests were used. Area under the curve graphs were generated using the integrated Prism formula without modification (baseline is considered *y* = 0). The specific type of statistical tests with exact *n* values and *P* values are indicated in the figures and legends, and further details are included in Supplementary Table 3. Unless stated otherwise, statistical significance was accepted at *P* < 0.05, and the exact *P* values are included in the graphs. In cases where no statistical difference was found between more than two groups, the overall analysis of variance (ANOVA) *P* value is presented.

### Reporting Summary

Further information on research design is available in the Nature Research Reporting Summary linked to this article.

## Online content

Any methods, additional references, Nature Research reporting summaries, source data, extended data, supplementary information, acknowledgements, peer review information; details of author contributions and competing interests; and statements of data and code availability are available at 10.1038/s41593-021-00923-4.

## Supplementary information

Supplementary Information Supplementary Figs. 1–6.

Reporting Summary

Supplementary Tables Table 1. hiPSC line sources and details. Table 2. List of antibodies used. Table 3. Details of statistical tests used and sample sizes.

Supplementary Data 1 DEGs by cell type for H-L1/H-L2 versus C9-L1 ALI-COs.

Supplementary Data 2 DEGs by cell type for ISO-L2 versus C9-L2 ALI-COs.

Supplementary Data 3 Top 25 GO terms per cell type for C9-L1 and C9-L2 ALI-COs.

Supplementary Data 4 Significant WGCNA module eigengenes per cell type in C9-L1 and C9-L2 ALI-COs.

Supplementary Data 5 Putative TF activity (defined by SCENIC) per cell type in C9-L1 and C9-L2 ALI-COs.

Supplementary Video 1 Virtual *z*-stack sectioning of a cleared H-L1 ALI-CO slice showing HOPX^+^, CTIP2^+^ and SATB2^+^ cell populations.

Supplementary Video 2 Virtual *z*-stack sectioning of a cleared H-L2 ALI-CO slice showing HOPX^+^, CTIP2^+^ and SATB2^+^ cell populations.

Supplementary Video 3 Virtual *z*-stack sectioning of a cleared C9-L1 ALI-CO slice showing HOPX^+^, CTIP2^+^ and SATB2^+^ cell populations.

Supplementary Video 4 Virtual *z*-stack sectioning of a cleared C9-L2 ALI-CO slice showing HOPX^+^, CTIP2^+^ and SATB2^+^ cell populations.

Supplementary Video 5 Virtual *z*-stack sectioning of a cleared ISO-L2 ALI-CO slice showing HOPX^+^, CTIP2^+^ and SATB2^+^ cell populations.

## Data Availability

Human cortical organoid scRNA-seq data have been deposited in the Gene Expression Omnibus database under accession code GSE180122. The experimental data that support the findings of this paper are provided as source data and in the Supplementary Information or are available from the corresponding authors upon request.

## Source data

Source Data Fig. 1 Unprocessed WB images (luminescence channel) and overlays with the molecular weight marker images (normal light channel).
Source Data Fig. 4 Unprocessed WB images (luminescence channel) and overlays with the molecular weight marker images (normal light channel).
Source Data Fig. 6 Unprocessed WB images (luminescence channel) and overlays with the molecular weight marker images (normal light channel).
Source Data Extended Data Fig. 1 Unprocessed WB images (luminescence channel) and overlays with the molecular weight marker images (normal light channel).
Source Data Extended Data Fig. 4 Unprocessed WB images (luminescence channel) and overlays with the molecular weight marker images (normal light channel).
Source Data Extended Data Fig. 6 Unprocessed WB images (luminescence channel) and overlays with the molecular weight marker images (normal light channel).

## Code availability

No new codes were generated in this work, and software availability is indicated in the Nature Research Reporting Summary.
