# Supplementary material for: Human ALS/FTD brain organoid slice cultures display distinct early astrocyte and targetable neuronal pathology
Source: Nat Neurosci. 2021 Oct 21;24(11):1542–54. doi: 10.1038/s41593-021-00923-4 (PMC8553627; doi:10.1038/s41593-021-00923-4)
Supplement: Supplementary file 2 — Reporting Summary [file 41593_2021_923_MOESM2_ESM.pdf]

nature research

Corresponding author(s): Dr András Lakatos and Dr Gabriel Balmus

Last updated by author(s): 15 July 2021

Reporting Summary

Nature Research wishes to improve the reproducibility of the work that we publish. This form provides structure for consistency and transparency in reporting. For further information on Nature Research policies, see our Editorial Policies and the Editorial Policy Checklist.

Statistics

For all statistical analyses, confirm that the following items are present in the figure legend, table legend, main text, or Methods section.

n/a	Confirmed	
☐	☒	The exact sample size (*n*) for each experimental group/condition, given as a discrete number and unit of measurement
☐	☒	A statement on whether measurements were taken from distinct samples or whether the same sample was measured repeatedly
☐	☒	The statistical test(s) used AND whether they are one- or two-sided<br>*Only common tests should be described solely by name; describe more complex techniques in the Methods section.*
☐	☒	A description of all covariates tested
☐	☒	A description of any assumptions or corrections, such as tests of normality and adjustment for multiple comparisons
☐	☒	A full description of the statistical parameters including central tendency (e.g. means) or other basic estimates (e.g. regression coefficient) AND variation (e.g. standard deviation) or associated estimates of uncertainty (e.g. confidence intervals)
☐	☒	For null hypothesis testing, the test statistic (e.g. *F*, *t*, *r*) with confidence intervals, effect sizes, degrees of freedom and *P* value noted<br>*Give P values as exact values whenever suitable.*
☒	☐	For Bayesian analysis, information on the choice of priors and Markov chain Monte Carlo settings
☒	☐	For hierarchical and complex designs, identification of the appropriate level for tests and full reporting of outcomes
☒	☐	Estimates of effect sizes (e.g. Cohen's *d*, Pearson's *r*), indicating how they were calculated

Our web collection on statistics for biologists contains articles on many of the points above.

Software and code

Policy information about availability of computer code

Data collection	MEA data collection was assisted by MC Data Tool, RRID: SCR_014580, https://www.multichannelsystems.com/software/mc-datatool
Data analysis	Established open source softwares, plugins or pipelines were used for data analysis, and the details have been provided in the manuscript and also below with full references and/or web links for accessibility. No new codes were developed for data analysis in this study.<br><br>CellProfiler Image Analysis Software, 3.1. 9 and 4.0.7, RRID: SCR_007358, https://cellprofiler.org/<br>CellRanger v3.1, https://support.10xgenomics.com/single-cell-gene-expression/software/release-notes/3-0<br>FIJI (ImageJ), 2.0.0, RRID: SCR_002285, https://fiji.sc/<br>ImageJ (OpenComet Plugin) SRC V1, RRID:SCR_001935, https://fiji.sc/<br>IncuCyte® NeuroTrack, RRID:SCR_019874, https://www.essenbioscience.com/en/products/peripherals/cell-player-neurotrack-software-module/<br>GraphPad, Version 7.0 or 8.0, RRID:SCR_002798, https://www.graphpad.com/<br>MATLAB, RRID: SCR_001622, http://www.mathworks.com/products/matlab/<br>Monocle3 0.2.3.0 - https://github.com/cole-trapnell-lab/monocle3<br>MC Data Tool, RRID: SCR_014580, https://www.multichannelsystems.com/software/mc-datatool<br>Python 3.6, https://www.python.org/downloads/<br>R version 4.0.3, RRID:SCR_001905, https://www.r-project.org<br>Seurat 3.1 and 4.0.1, https://github.com/satijalab/seurat/<br>Scmap 1.12.0 , https://github.com/hemberg-lab/scmap<br>SCENIC 0.9.6 https://github.com/aertslab/SCENIC<br>WGCNA 1.70-3, http://horvath.genetics.ucla.edu/html/CoexpressionNetwork/Rpackages/WGCNA/

For manuscripts utilizing custom algorithms or software that are central to the research but not yet described in published literature, software must be made available to editors and reviewers. We strongly encourage code deposition in a community repository (e.g. GitHub). See the Nature Research guidelines for submitting code & software for further information.

Data

Policy information about availability of data

All manuscripts must include a data availability statement. This statement should provide the following information, where applicable:

- Accession codes, unique identifiers, or web links for publicly available datasets
- A list of figures that have associated raw data
- A description of any restrictions on data availability

1. scRNA-seq data that support the findings have been deposited in the GEO database (accession code: GSE180122).
2. The figures with associated raw data (GEO and Supplementary Data Files 1-5) include Figures 1-3, Extended Data Figures 1-3, Supplementary Figure 3.
3. Data is also available from the corresponding authors.

Field-specific reporting

Please select the one below that is the best fit for your research. If you are not sure, read the appropriate sections before making your selection.

☒ Life sciences ☐ Behavioural & social sciences ☐ Ecological, evolutionary & environmental sciences

For a reference copy of the document with all sections, see nature.com/documents/nr-reporting-summary-flat.pdf

Life sciences study design

All studies must disclose on these points even when the disclosure is negative.

Sample size	Statistical test details and sample sizes have been summarised in Supplementary Table 3. No sample size calculation was performed. The sample sizes using hiPSC lines and organoids were estimated from previously performed experiments (e.g. Giandomenico et al., Nature Neuroscience (2019); Tyzack et al., Nature communications (2017). Sample sizes and origins are summarized in Supplementary Table 1 and 3.
Data exclusions	No acquired data was excluded from the statistical analyses. (A few damaged sections/slices were not used for data collection).
Replication	Experiments were repeated three times (or two times for GSK treatments), which included at least 3 independent biological replicates (organoids/cells grown from separate independent batches and/or cell lines), leading to similar results. At least three biological replicates were used for all statistical analyses in biological experiments.
Randomization	The in vitro experiments were not fully randomized. Sample allocations into groups included independent organoids, ALI-COs or immersed CO slices grown from different cell lines and/or as separate batches (independent biological replicates). Batches of organoids were randomly selected from each cell line-group. ALI-COs or immersed CO slices deriving from identical whole organoids were only used in separate studies or as adequate control-treatment slice-pairs for each independent biological replicate. No randomization was used for cell culture experiments using non-differentiated cultures of hiPSCs.
Blinding	For analysis the subjects were blinded for the observers. This involved masking original sample identification and assigning coded IDs before data collection.

Reporting for specific materials, systems and methods

We require information from authors about some types of materials, experimental systems and methods used in many studies. Here, indicate whether each material, system or method listed is relevant to your study. If you are not sure if a list item applies to your research, read the appropriate section before selecting a response.

Materials & experimental systems

n/a	Involved in the study	
☐	☒	Antibodies
☐	☒	Eukaryotic cell lines
☒	☐	Palaeontology and archaeology
☒	☐	Animals and other organisms
☒	☐	Human research participants
☒	☐	Clinical data
☒	☐	Dual use research of concern

Methods

n/a	Involved in the study	
☒	☐	ChIP-seq
☒	☐	Flow cytometry
☒	☐	MRI-based neuroimaging

Antibodies

Antibodies used	The details of all antibodies are provided below with validation references, and are also listed in Supplementary Table 2.

April 2020

Validation

Antibody details, Catalogue number, validation references:
Rabbit anti-53BP1, Novus NB100-304
https://www.novusbio.com/products/53bp1-antibody_nb100-304
Mouse anti-ACTB-HRP, Proteintech HRP-60008
RRID:AB_2819183
Rabbit anti-AQP4, Millipore Sigma HPA014784
RRID:AB_1844967
Mouse anti-bIII-tubulin, Abcam ab78078
RRID:AB_2256751
Chicken anti-bIII-tubulin, Merck AB9354
RRID:AB_570918
Rat anti-CTIP2, Abcam ab18465
RRID:AB_2064130/ AB_10015215
Rabbit anti-EIF2a, New England Biolabs 9722
RRID:AB_2230924/AB_10695409
Rabbit anti-p-EIF2a, New England Biolabs 9721
RRID:AB_330951/AB_330952
Rabbit anti-FOXG1, Abcam ab18259
https://www.abcam.com/FOXG1-antibody-ab18259.html?gclsrc=aw.ds|aw.ds&gclid=CjwKCAjw55-HBhAHEiwARMCszubMZRSiz-G0r2DZe4OZnl7_SIDmRsM9kjgWutq9Jl66pcXN3e9HShoC2CMQAvD_BwE
Mouse anti-GAD65, Abcam ab26113
https://pubmed.ncbi.nlm.nih.gov/31298263/
Mouse anti-GFAP, Millipore Sigma G6171
RRID:AB_1840893
Rabbit anti-GFAP, Abcam ab7260
https://www.abcam.com/gfap-antibody-ab7260.html
Chicken anti-GFAP, Antibodies.com A85307
RRID:AB_2748894
Mouse anti-γ-H2AX, Millipore 05-636
RRID:AB_309864
Mouse anti-HepaCAM, R&D Systems MAB4108
RRID:AB_2117687
Rabbit anti-HOMER1, Synaptic Systems Cat# 160 003
RRID:AB_887730
Rabbit anti-HOPX, Millipore Sigma HPA030180
RRID:AB_10603770
Rabbit anti-LC3, Abcam ab192890
RRID:AB_2827794
Chicken anti-MAP2, Abcam ab5392
https://www.abcam.com/map2-antibody-ab5392.html
Mouse anti-NESTIN, Abcam ab22035
RRID:AB_446723
Rabbit anti-NEUN, Millipore Sigma ABN78
RRID:AB_10807945
Mouse anti-NEUN, Abcam ab104224
RRID:AB_10711040
Rabbit anti-PABP1, Cell Signaling Technology 4992
RRID: AB_10693595/AB_2156887
Rabbit anti-POLY(GA), Proteintech 24492-1-AP
RRID:AB_2879571
Mouse anti-SATB2, Abcam ab5150
RRID:AB_91701
Goat anti-SOX2, Santa Cruz sc-17320
https://pubmed.ncbi.nlm.nih.gov/24178749/
Rabbit anti-SOX9, Millipore Sigma AB5535
RRID:AB_2239761
Mouse anti-SQSTM1/P62, Abcam ab5641
RRID:AB_2747598
Mouse anti-SYT1, Synaptic Systems 105 011
RRID:AB_2619761
Goat Anti-Rabbit IgG-HRP, ThermoFisher 31462
RRID:AB_228338
Goat Anti-Mouse IgG-HRP, Vector Laboratories Inc. PI-2000
https://vectorlabs.com/peroxidase-horse-anti-mouse-igg-antibody.html#biozbadges
Donkey Anti-Goat Alexa Fluor-488 , ThermoFisher A11055
RRID:AB_2534102
Goat Donkey Anti-Mouse Alexa Fluor-568, ThermoFisher A10037
RRID:AB_2534013
Goat Anti-Mouse Alexa Fluor-568 , ThermoFisher A11031
RRID:AB_144696
Goat Anti-Rabbit Alexa Fluor-568, ThermoFisher A11036
RRID:AB_10563566
Goat Anti-Rabbit Alexa Fluor-488, ThermoFisher A11008
RRID:AB_143165
Goat Anti-Mouse Alexa Fluor-488, ThermoFisher A11029

RRID:AB_2534088
Goat Anti-Rat Alexa Fluor-568, Abcam ab175710
RRID:AB_2832918
Goat Anti-Chicken Alexa Fluor-647, ThermoFisher A32933
RRID:AB_2762845
Goat Anti-Rabbit Alexa Fluor-647, Abcam ab150083
RRID: AB_2714032

Eukaryotic cell lines

Policy information about cell lines

Cell line source(s)	All cell line sources and details are listed in Supplementary Table 1.
Authentication	The genotype/hexanucleodite repeat length were tested and cross-checked for the cell lines against the information provided by the company. The Southern blot image and PCR fragment analysis are included in Extended Data Figure 1.
Mycoplasma contamination	Media from cell lines were randomly tested for Mycoplasma in our laboratory, and there has been no evidence for contamination by Mycoplasma.
Commonly misidentified lines (See ICLAC register)	No commonly misidentified cell lines were used.
