# Supplementary material for: Human ALS/FTD brain organoid slice cultures display distinct early astrocyte and targetable neuronal pathology
Source: Nat Neurosci. 2021 Oct 21;24(11):1542–54. doi: 10.1038/s41593-021-00923-4 (PMC8553627; doi:10.1038/s41593-021-00923-4)
Supplement: Source Data Extended Data Fig. 4 — Unprocessed WB images (luminescence channel) and overlays with the molecular weight marker images (normal light channel). [file 41593_2021_923_MOESM18_ESM.pdf]

c MARKER (M) OVERLAY UNCROPPED BLOT

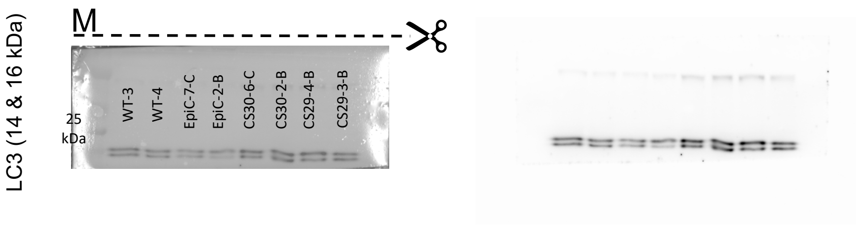

Source Data Extended Data Figure 4 | Uncropped western blot (WB) scans (right; luminescence channel) and their overlays with molecular weight markers (left; light channel) for Extended Data Fig. 4c. Sample labels refer to hiPSC-derived organoid line codes (see Supplementary Table 1) followed by batch codes. Dashed lines indicate blot membrane cuts that enabled simultaneous detection of multiple proteins in the same sample.
