# Supplementary material for: Human ALS/FTD brain organoid slice cultures display distinct early astrocyte and targetable neuronal pathology
Source: Nat Neurosci. 2021 Oct 21;24(11):1542–54. doi: 10.1038/s41593-021-00923-4 (PMC8553627; doi:10.1038/s41593-021-00923-4)
Supplement: Supplementary file 1 — Supplementary Figs. 1–6. [file 41593_2021_923_MOESM1_ESM.pdf]

nature portfolio

https://doi.org/10.1038/s41593-021-00923-4

Supplementary information

Human ALS/FTD brain organoid slice cultures display distinct early astrocyte and targetable neuronal pathology

In the format provided by the authors and unedited

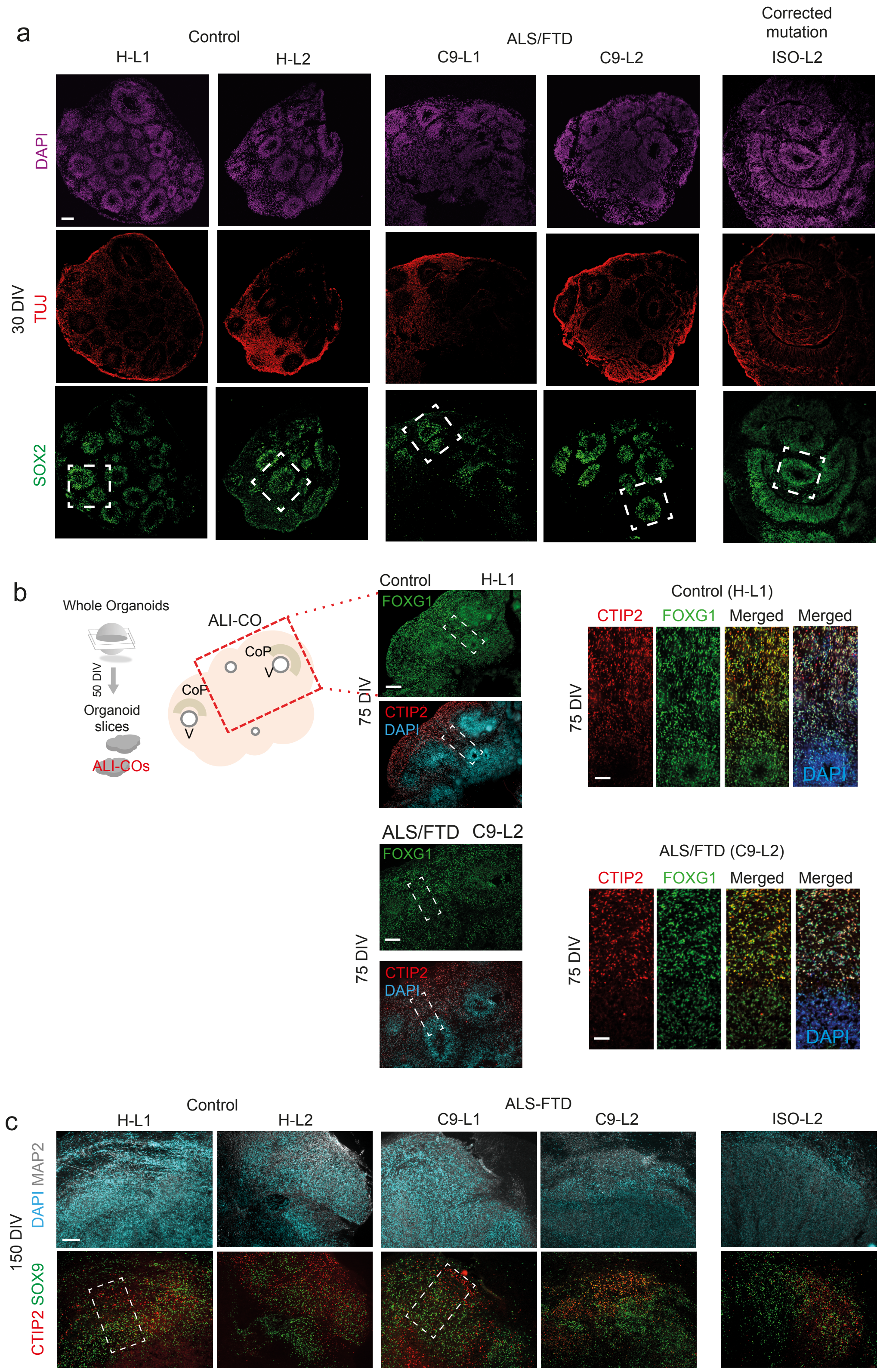

Supplementary Figure 1 | Early and late organoid formation. a, Representative low magnification immunofluorescence images demonstrating palisade-like organization of SOX2+ progenitor cells (SOX2, green) surrounded by immature neurons (TUJ1, red) in cortical organoids at 30 DIV for 3 biological repeats per group (source for Extended Data Fig. 1d). **b,** Schematic (left) and representative immunofluorescence images (middle) with insets (right) of cortical plate (CoP) areas at 75 DIV, displaying forebrain identity marker FOXG1- (green), deep cortical layer neuron (DLN) marker CTIP2-(red)-immunolabelling and DAPI staining from 3 biological repeats per group. **c,** Source images for Fig. 1e showing immunostained ALI-COs at 150 DIV, displaying CTIP2+ DLNs (red) and SOX9+ astroglia (green) with or without DAPI-stain (cyan) and MAP2+ neurons (grey) for 3 biological repeats per group. Scale bar=100 μm for a, 150 μm for b (left panel) and 40 μm for b (right panel) and 80 μm for c.

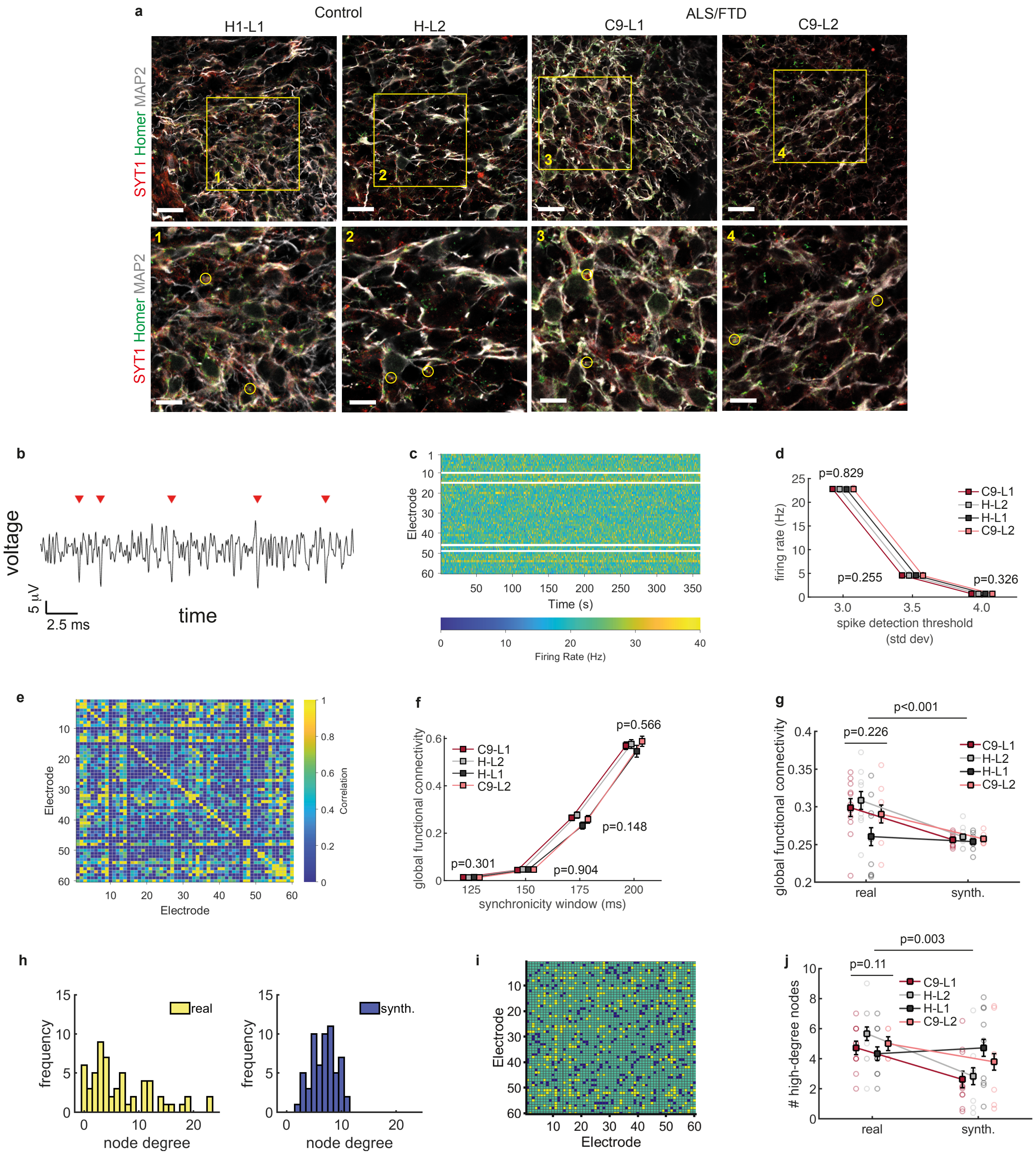

Supplementary Figure 2 | Astroglia and neuronal subtypes, displaying circuit activity in control and C9 ALI-COs. **a,** Representative large views (upper panel) and magnified areas (insets 1-4) of confocal microscopy images illustrate pre- and post-synaptic proteins, synaptotagmin-1 (SYT1, red) and Homer1 (green) and also their colocalization over MAP2+ dendrites (gray) for ALI-COs at 150 DIV. n=4,6,6,5 independent ALI-CO slices for H-L1, H-L2, C9-L1, C9-L2 organoid lines, respectively (supplementary images for Fig. 2e). **b-j,** Source data for Fig 2f,g: **b,** Sample trace with spikes detected (red triangles) using the mean minus three standard deviations as the threshold. **c,** Temporal rasterplot from a single recording showing firing rate over the 6-minute-long recording (1 s time bins). **d,** Plot shows firing rate by cell line for three different spike detection thresholds based on the standard deviation of voltage fluctuations in the electrodes. **e,** Correlation matrix using the spike-timing tiling coefficient (STTC) from the same recording in B used to estimate global connectivity, node degree, and edge weight. **f,** Spike-timing tiling coefficient by cell line for four synchronicity windows. **g,** Global functional connectivity in the real networks by cell line compared with synthetic spike matrices for each recording (created with matched spike trains for each electrode in the recording). **h,** Distribution of the node degree from a single recording (left) compared against a surrogate graph (right) created based on the synthetic spike matrix for the recording. **i,** Sample comparison of the binary connection matrices of real and surrogate network. Yellow indicates a connection present in the real network but not the surrogate; blue indicates the reverse. **j,** The number of high-degree nodes (≥30 connections) in the real networks by cell-line compared with synthetic spike matrices for each recording. Data expressed as mean±s.e.m.; n=9,9,11,6 slice recordings; One-way ANOVA for c, e. with no posthoc test, and Two-way ANOVA for f,i. with Welch's t-test for multiple group comparisons. Scale bar=20 μm (upper panel) and 10 μm (lower panel) in a. See Supplementary Table 3 for detailed statistics.

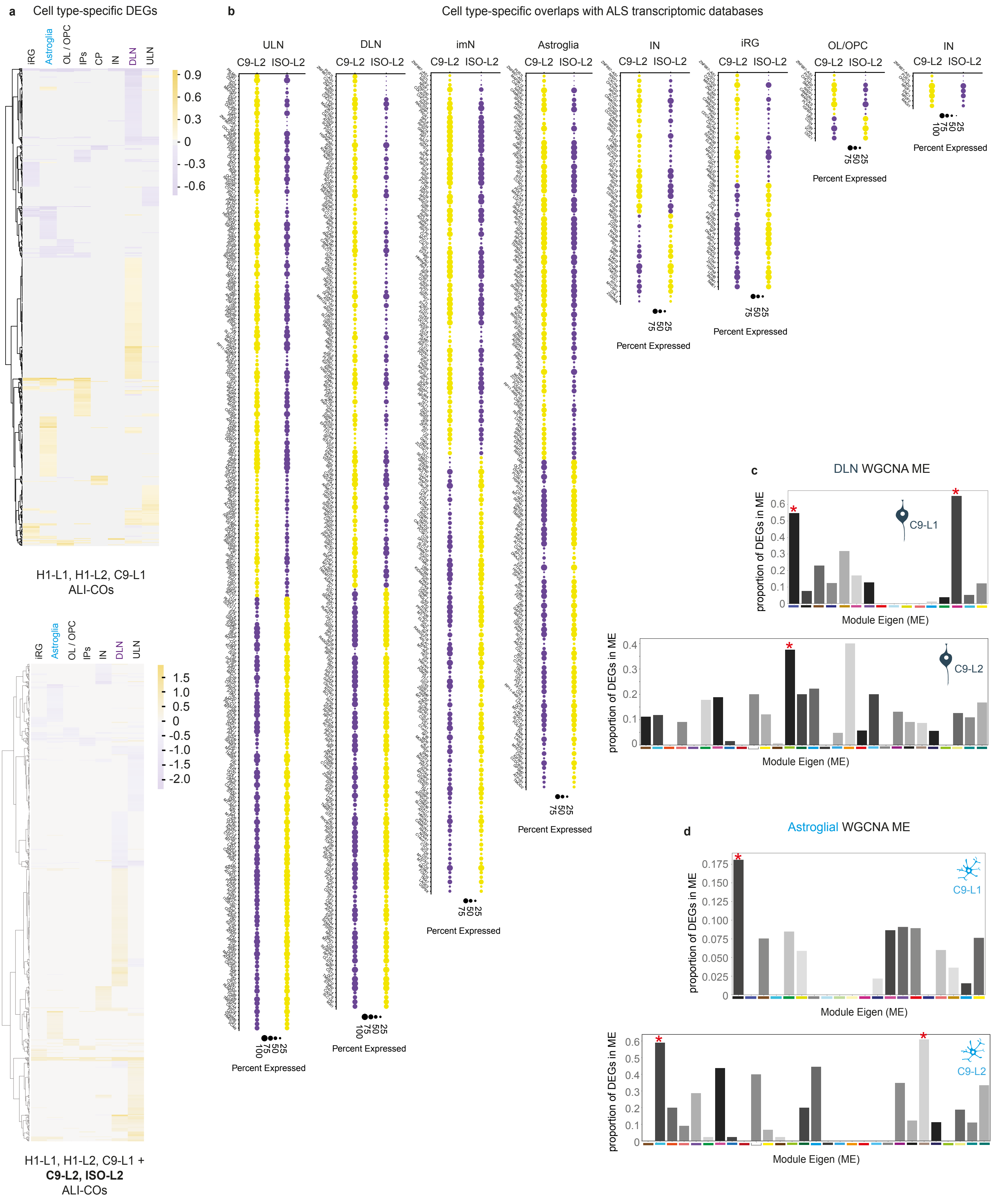

Supplementary Figure 3 | Cell-type-specific transcriptional profiles of C9 ALI-COs and their overlaps with ALS patient-related gene expression data. a, Heatmaps demonstrating log-fold change in differentially expressed genes (DEGs; adjusted p-value <0.05) between controls and C9 ALI-COs at 150 DIV, grouped by major cell types (supplementary data for Fig.3a,b). **b,** Dot plots represent the overlap in transcriptomic changes between cell types in C9-L2 ALI-COs, isogenic mutation-corrected control ISO-L2 ALI-COs and human ALS patient samples[20–25]. Dot size indicates the percentage of cells expressing upregulated (yellow) and downregulated (purple) genes (supplementary data for Fig. 3c and Extended Data Fig. 3a). **c** and **d,** Bar plots showing the proportion of DEGs for either deep layer neurons (DLN; c) or astroglia (d) within each Weighted Gene Co-expression Network Analysis (WGCNA) Module Eigen (ME). Asterisks represent significant modules with the highest DEG proportions (supplementary data for Fig. 3f and Extended Data Fig. 3c).

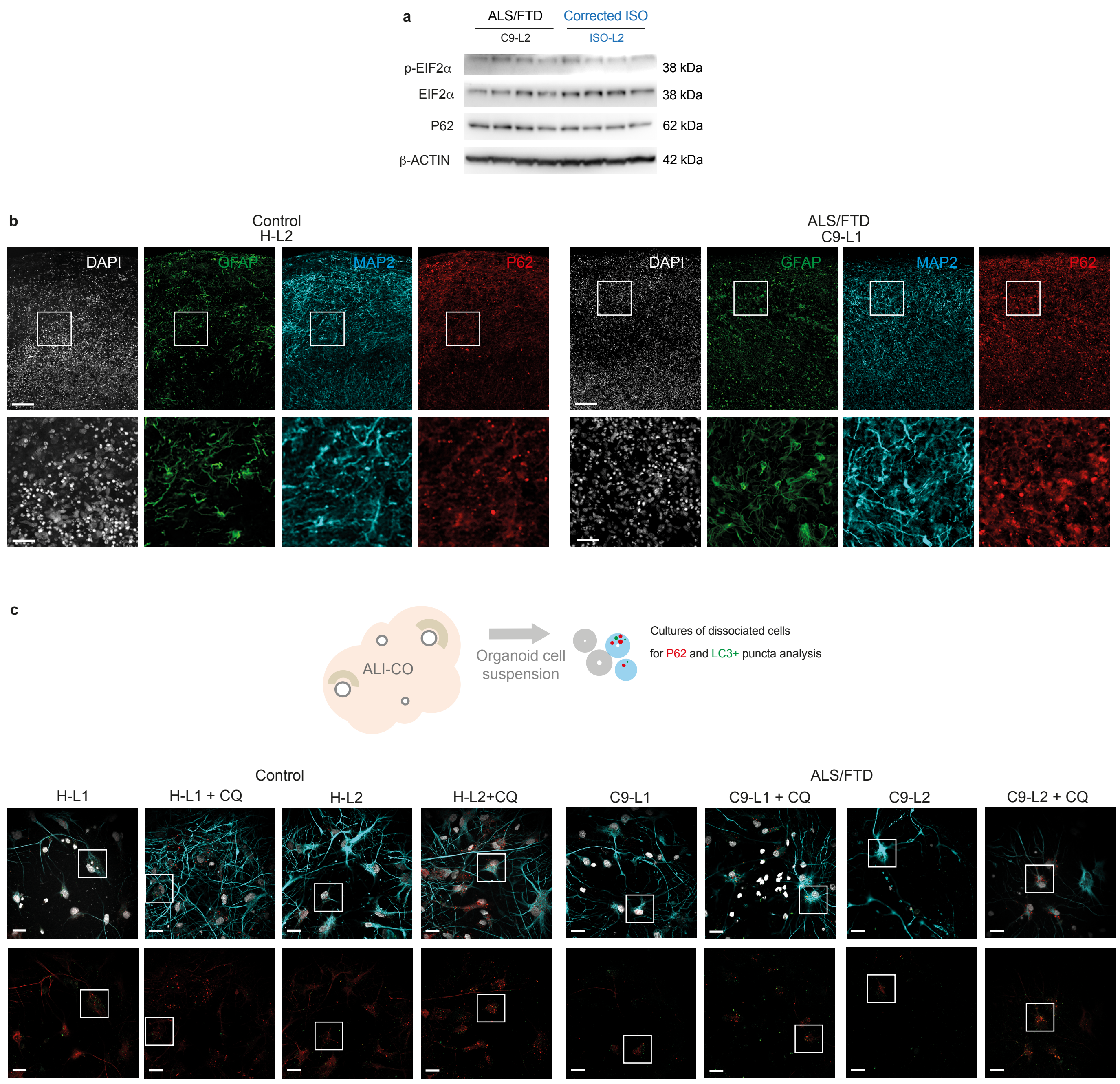

Supplementary Figure 4 | Autophagy is activated in C9 ALI-COs and astroglia. a, Representative western blots (WBs) for ER-stress/unfolded protein response elements p-EIF2α/EIF2α, autophagy marker P62, and β-actin in 4 C9-L2 vs 4 ISO-L2 ALI-CO whole slice sample repeats per group (supplementary images for Fig. 4c; see Supplementary Fig. 6 for unprocessed WB images). **b,** Representative low magnification immunofluorescence images of healthy control and C9 ALI-COs at 150 DIV, displaying autophagy marker P62 (red), astroglial marker GFAP (green) and mature neuronal marker MAP2 (cyan) labelling for 3 biological repeats per group (source images for Extended Data Fig. 4a). **c,** Confocal microscopy images of dissociated cell cultures of control and C9 ALI-COs with p62 (red) and LC3 (green) autophagy marker immunolabeling with or without chloroquine (CQ) treatment in GFAP+ (cyan) astroglia for 4 experimental repeats per group (source images for Extended Data Fig. 4b). Scale bar: 80 μm (upper panel) and 20 μm (lower panel) for all channels in b, and 20 μm for c.

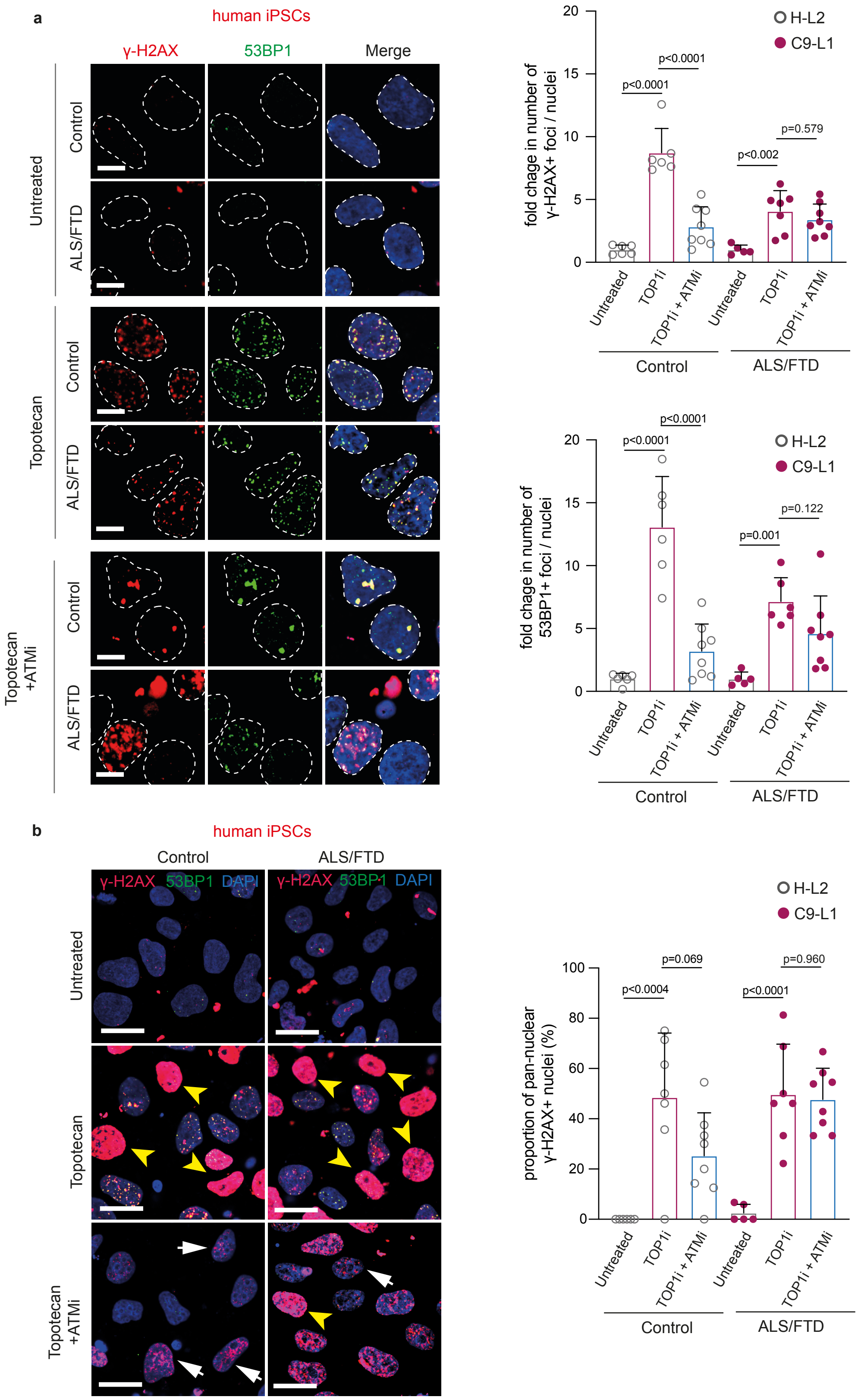

Supplementary Figure 5 | ATM-dependent DNA repair signaling is defective in C9 human iPSCs. **a,** Representative confocal microscopy immunofluorescence images displaying DNA damage marker γ-H2AX+ foci (red) and 53BP1+ foci (green) in control and C9 human iPSCs with or without TOP1i (10μM) treatment±ATM kinase inhibitor AZD0156 (ATMi; 30nM) and quantification. Quantification of DNA damage. Data expressed as fold change of γ-H2AX+ or 53BP1+ foci/nuclei and presented as mean±s.d.; n=6,6,8 or 5,7,8 culture repeats (>30 cells/replicate) per cell line for untreated, TOP1i treated and TOP1i±ATMi cells, respectively; One-way ANOVA with Tukey's multiple comparisons test. **b.** Representative confocal microscopy immunofluorescence images of control and C9 human iPSCs displaying 53BP1+ foci (green) and γ-H2AX+ pan-nuclear staining (red with yellow arrowheads) that indicates cell death versus DNA damage signaling for repair (white arrows)[43] in response to TOP1i±ATMi administration. Graph displays the proportion of pan-nuclear γ-H2AX+ foci/cell as mean±s.d.; n=5,7,8 culture repeats for untreated, TOP1i treated and TOP1i±ATMi cells (>30 cells/replicate) per cell line, respectively; One-way ANOVA with Tukey's multiple comparisons test. Size bar=5 μm for all channels in a, 10 μm for b. See Supplementary Table 3 for detailed statistics.

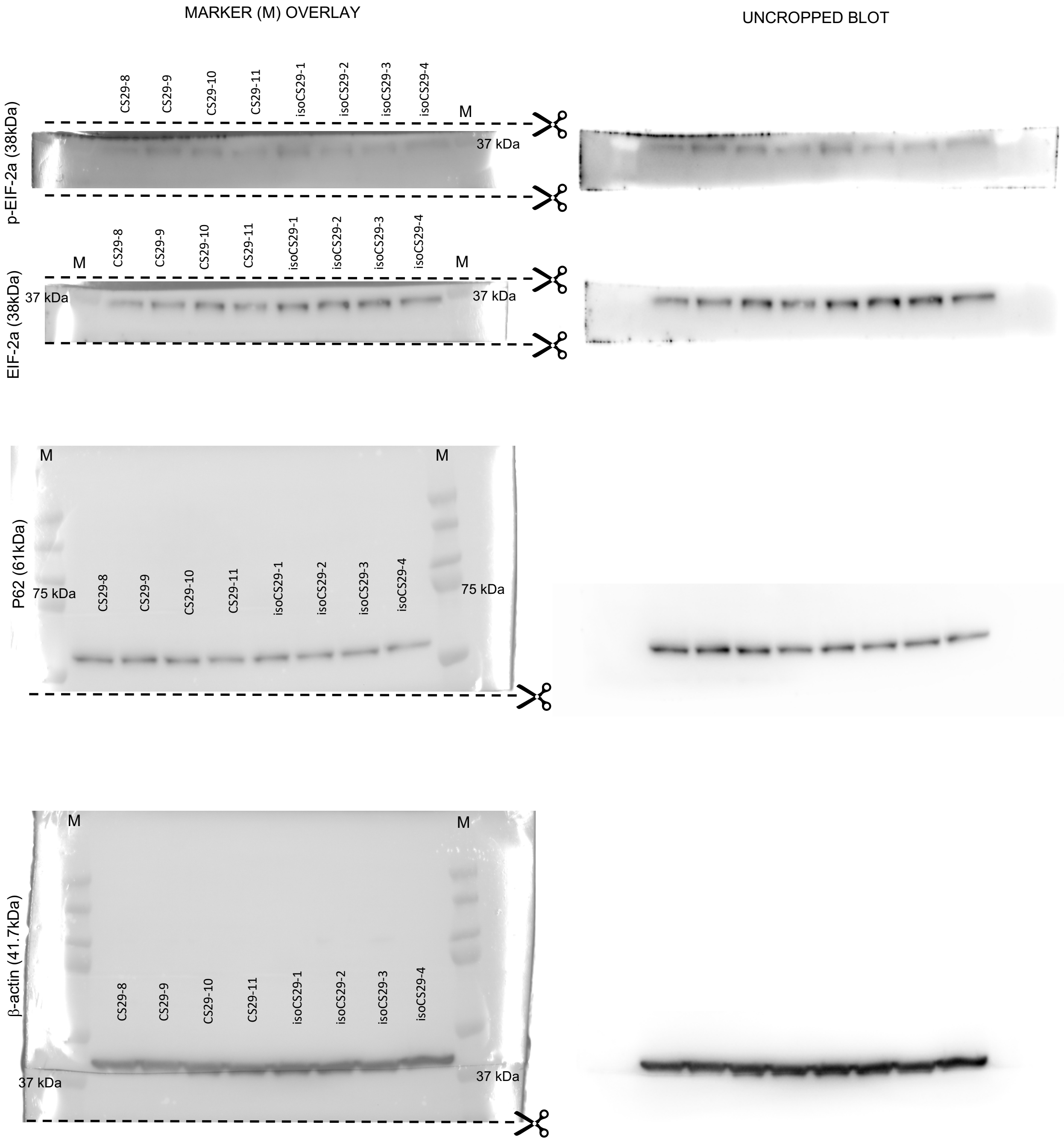

Supplementary Figure 6 | Uncropped western blot (WB) scans (right; luminescence channel) and their overlays with molecular weight markers (left; light channel) for Supplementary Fig. 4a. Sample labels refer to hiPSC-derived organoid line codes (see Supplementary Table 1) followed by batch codes. Dashed lines indicate blot membrane cuts that enabled simultaneous detection of multiple proteins in the same sample.
